# Tetraphenyl-1,4-dioxin and Tetraphenyl-pyrane-4-one: Old Molecules, New Insights

**DOI:** 10.1021/acsomega.3c01226

**Published:** 2023-05-22

**Authors:** Medine Soydan, Burcu Okyar, Yunus Zorlu, Antoine Marion, Salih Özçubukçu

**Affiliations:** † Department of Chemistry, Middle East Technical University, 06800 Ankara, Turkey; ‡ Department of Chemistry, Gebze Technical University, 41400 Kocaeli, Turkey

## Abstract

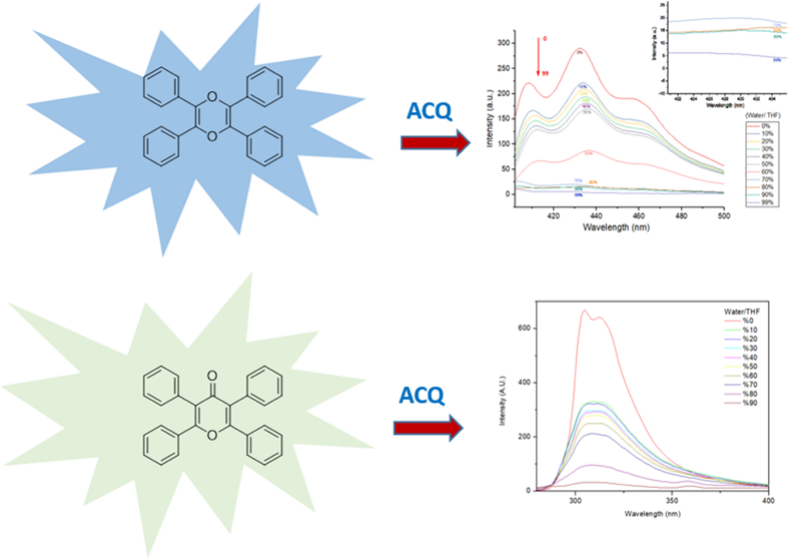

Aggregation-induced emission (AIE) is a phenomenon where certain molecules or materials become highly luminescent when they aggregate or come together in a condensed state, such as a solid or a solution. Moreover, new molecules which show AIE properties are designed and synthesized for various applications like imaging, sensing, and optoelectronics. 2,3,5,6-Tetraphenylpyrazine (TPP) is one of the well-established examples of AIE. Herein, 2,3,5,6-tetraphenyl-1,4-dioxin (TPD) and 2,3,4,5-tetraphenyl-4*H*-pyran-4-one (TPPO), which are old molecules with TPP similarity, were studied, and new insights in terms of structure and aggregation-caused quenching (ACQ)/AIE properties were gained by means of theoretical calculations. Those calculations performed on TPD and TPPO aimed to provide a better understanding of their molecular structures and how they affect their luminescence properties. This information could be used to design new materials with improved AIE properties or to modify existing materials to overcome ACQ.

## Introduction

**Figure 1:**
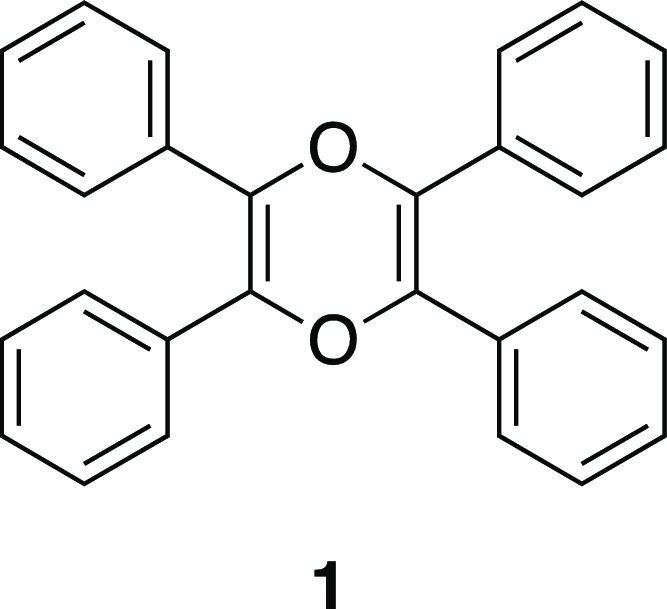
Structure of 2,3,5,6-tetraphenyl-1,4-dioxin (**1**).

Tetraphenyl derivative of 1,4-dioxin (2,3,5,6-tetraphenyl-1,4-dioxin) (**1**) drew the attention of many scientists decades ago. Dioxin **1** (Figure 1) has been known for decades, but in fact there were some conflicts about the synthesis of this molecule.^1^ In 1959, Berger and Summerbell performed the synthesis of **1**, by using benzoin as a starting material, followed by the addition of *p*-toluenesulfonic acid in catalytic amount. After some purification methods, it was obtained as a precipitate with an 8% yield.^2^ Later in 1964, Jerumanis and Lalancette came up with a new synthesis method for dioxin **1**, by using methyl benzoate as a starting material and continued with the addition of boron sulfide. The procedure was followed with some crystallization methods and claimed that dioxin **1** was obtained as a product.^3^ However, in 1978, Yager and Wootan tried to figure out the discrepancies in these two methods and realized that the actual synthesis of dioxin **1** was achieved by Berger and Summerbell, while Jerumanis and Lalancette synthesized tetraphenyl thiophene. This error was due to incorrect elemental analysis according to Yager and Wootan. In 1985, Schmidt et al. offered a new synthesis method for dioxin **1**, which consisted of two steps. The first step was the formation of mono- and di-methoxy-substituted dimers after bubbling of HCl gas in dry methanol and the second step was the formation of the crystals of dioxin **1** after refluxing in acetic anhydride in the presence of *p*-TsOH.^4^ Characterization of dioxin **1** was performed by only elemental analysis and melting point. There is no NMR data reported for this known heterocycle in the literature.

**Figure 2:**
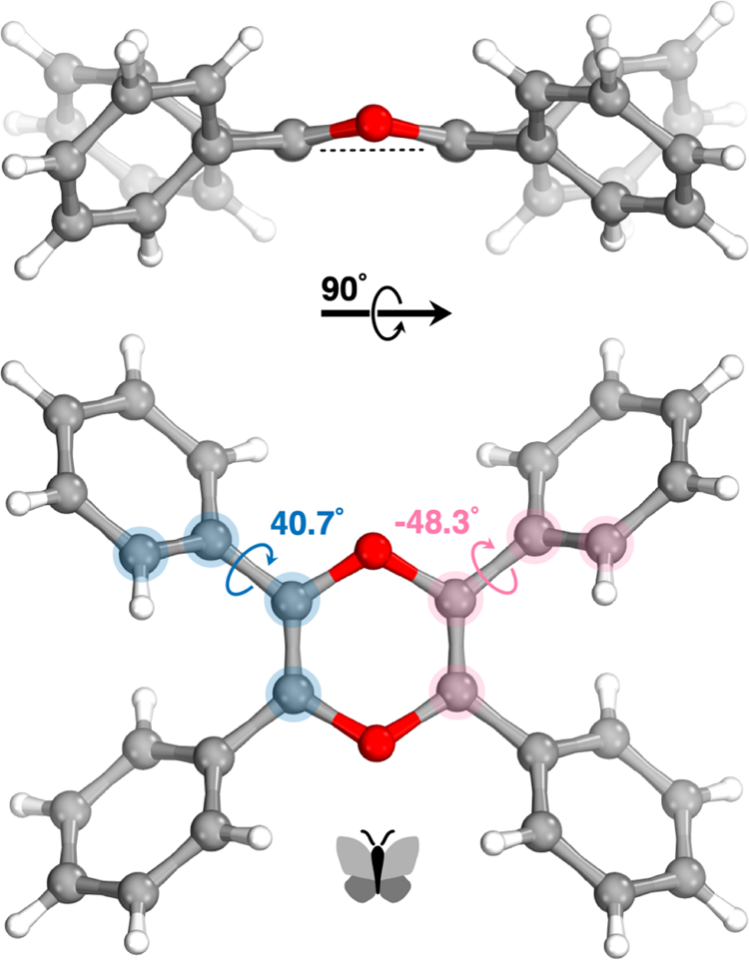
Molecular structure of dioxin **2** and **3**.

In 2000, Mamedov and his co-workers investigated the structure of some similar dioxin derivatives by X-ray analysis.^5^ They studied two dioxin derivatives: dimethyl 2,5-diphenyl-1,4-dioxine-3,6-dicarboxylate (**2**) and dimethyl 2,6-diphenyl-1,4-dioxine-3,5-dicarboxylate (**3**) (Figure 2). Albeit being regioisomers of each other, these two dioxin derivatives show two very different geometries for the central dioxin ring. Dioxin **2** has a planar dioxin core, whereas dioxin **3** has a boat conformation. Both phenyl rings in dioxin **2** and **3** are perpendicular to the core rings. The double bonds in the dioxin ring of **3** are shorter than they are in **2**. The conjugation in dioxin **2** is more likely among the planar dioxin core, the carbonyl group of the methyl ester group, and the lone pairs of “O” atom in the endocyclic ring, whereas the conjugation in dioxin **3** is infeasible due to the nonplanar structure of dioxin. Moreover, the crystal structure of sulfur analogue of dioxin **1**; 2,3,5,6-tetraphenyl-1,4-dithiin has been studied by Hoggard and Jones.^6^ They found that phenyl rings are all neither perpendicular nor parallel to the central ring. Instead, it is a mixture of these orientations and the dithiin ring has a boat conformation similar to unsubstituted 1,4-dithiin.

## Results and Discussion

Being so much different in geometry depending on the substituents and heteroatom in the central ring, the structure of dioxin **1** drew our attention, and herein we report the full characterization and crystal structure of dioxin **1** and its fluorescence properties due to the similarity of its structure to another heterocyclic ring system, that is, pyrazine.

We followed the synthetic method of Schmidt and his co-workers and obtained the dioxin **1** with an overall yield of 18% after two steps as yellow solids. It was further crystallized in a toluene and petroleum ether mixture.

**Figure 3:**
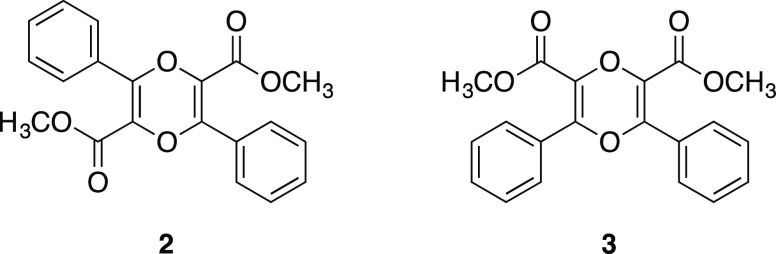
Crystal structure of dioxin **1** showing the dihedral angles of the phenyl rings.

Compound **1** lies across a crystallographic inversion center and its six-membered central dioxin core has a quite flattened boat conformation with a total puckering amplitude *Q*_T_ of 0.225(13) Å and the phenyl rings are positioned as the wings of a butterfly, as shown in Figure 3. Two crystallographically independent phenyl rings form 40.7° and −48.3° dihedral angles with the dioxin core ring. The C=C double bond in the dioxin core has almost the same length (1.329 Å) as the double bond in the cyclohexene (1.326 Å). This result implies that lone pairs of oxygen in the dioxin ring is not resonating with the double bond. The same bond length in dioxin **3** (CSD refcode: QEVGIH) is reported^5^ as 1.332–1.335 Å, which is almost the same as that of dioxin **1**. Also, the endocyclic O–C bond lengths (1.393–1.413 Å) in the dioxin core of **1** are nearly similar (1.393–1.402 Å) to those of **3**. ^1^H and ^13^C NMR spectra of dioxin **1** were also analyzed. The carbon peak in the dioxin core ring is resonated at 136 ppm. This also suggests that the dioxin **1** core is not aromatic.

The aggregation-induced emission (AIE) phenomenon is known as the increase in emission intensity when molecules with this property aggregate or are in solid form. It was proposed that AIE takes place due to the restriction of intramolecular motions (RIMs) which are restriction of intramolecular rotation (RIR) and restriction of intramolecular vibration (RIV).^7^ Molecules with this property have a butterfly/propeller shape and are composed of olefinic or aromatic stators with multiple phenyl rotors. Also, phenyl groups rotate nearly freely around a single bond, which is claimed to be the way in which the molecules relax in solution nonradiatively. As molecules with AIE properties aggregate, the free rotation and intramolecular vibration are prevented, and thus the AIE phenomenon is observed. Moreover, the AIE process is promoted in those molecules since the geometry does not allow *π*–*π* stacking, which is a relaxation path for excited molecules.^8^ 2,3,5,6-Tetraphenylpyrazine has been known in the literature for a long time, and recently its aggregation-induced emission (AIE) property has been investigated along with other para-substituted derivatives by Tang et al.^9^

**Figure 4:**
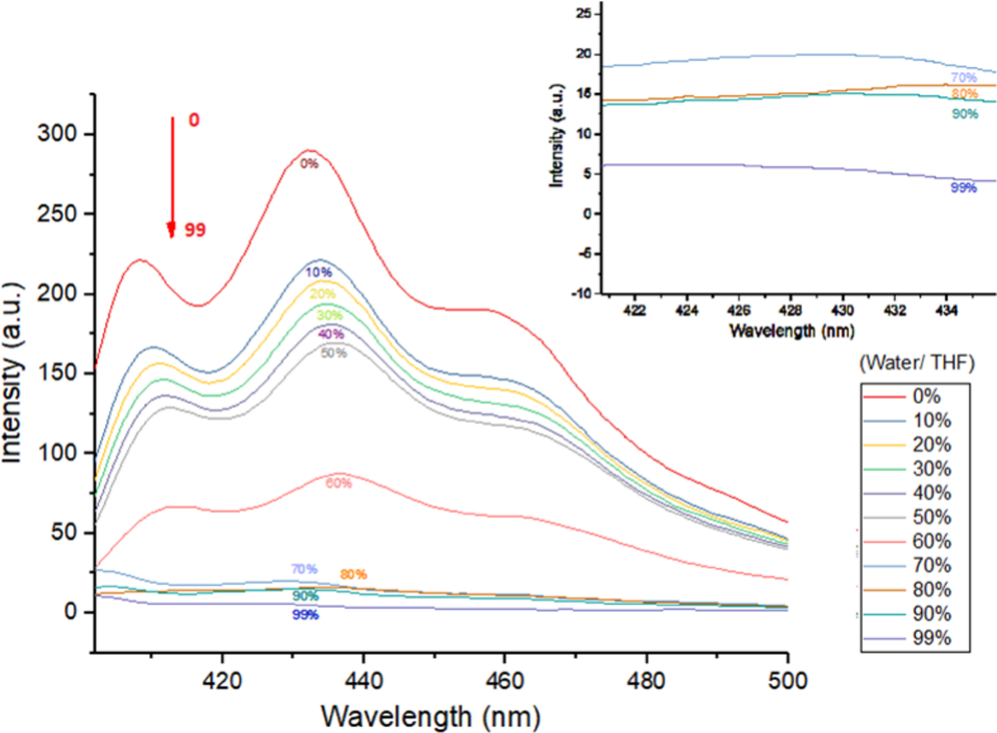
Fluorescence spectrum of 2,3,5,6-tetraphenyl dioxin (dioxin **1**) in a THF/H_2_O mixture.

Dioxin **1** has a structure similar to that of 2,3,5,6-tetraphenylpyrazine. We therefore decided to investigate the aggregation-induced emission character of dioxin **1**. The fluorescence spectrum of dioxin **1** in pure tetrahydrofuran (THF) and in a THF/H_2_O mixture has been studied carefully (Figure 4), and interestingly, dioxin **1** showed aggregation-caused quenching (ACQ) rather than AIE.

**Figure 5:**
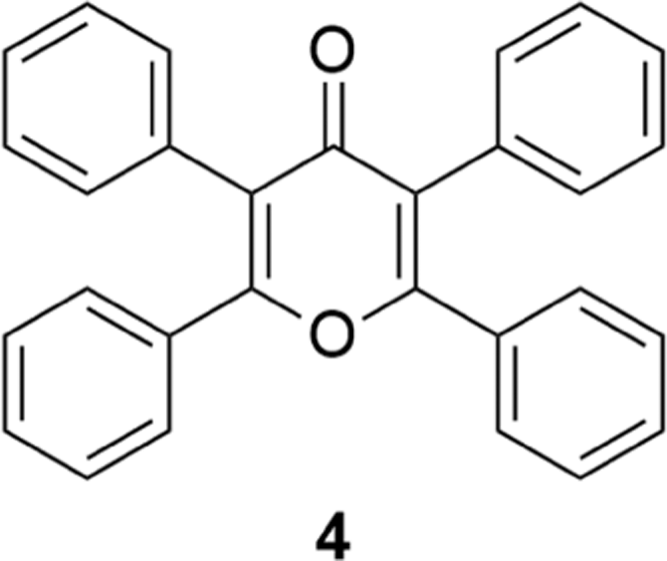
Structure of 2,3,5,6-tetraphenyl-4*H*-pyran-4-one (pyranone **4**).

After these results, we synthesized 2,3,5,6-tetraphenyl-4*H*-pyran-4-one, which is pyranone **4** (Figure 5), as a potentially planar counterpart of dioxin **1**. Pyranone **4** is less electron-rich compared to dioxin **1**, but it has an aromatic core. We aimed to investigate how these changes would affect the indication of ACQ/AIE property of 2,3,5,6-tetraphenyl-4*H*-pyran-4-one (pyranone **4**).

There are two methods to synthesize the pyranone **4**. In 1959, Friedrich and Bernhauer synthesized pyranone **4** with benzoic anhydride and boric acid at 250 °C in one step.^10^ Yates and Weisbach in 1963 obtained pyranone **4** with the reaction of deoxybenzoin and phosgene in toluene under reflux in 75 min in one pot with 8.5% yield.^11^ When Friedrich and Bernhauer’ synthesis is considered, the reaction conditions are notably harsh. Also, Yates and Weisbach employed phosgene which is highly toxic, and the yield of the reaction is considerably low. Moreover, pyranone **4** was poorly characterized. Only elemental analysis, melting point analysis, and infrared (IR) spectrum of the pyranone **4** are available in the literature.

**Scheme 1:**
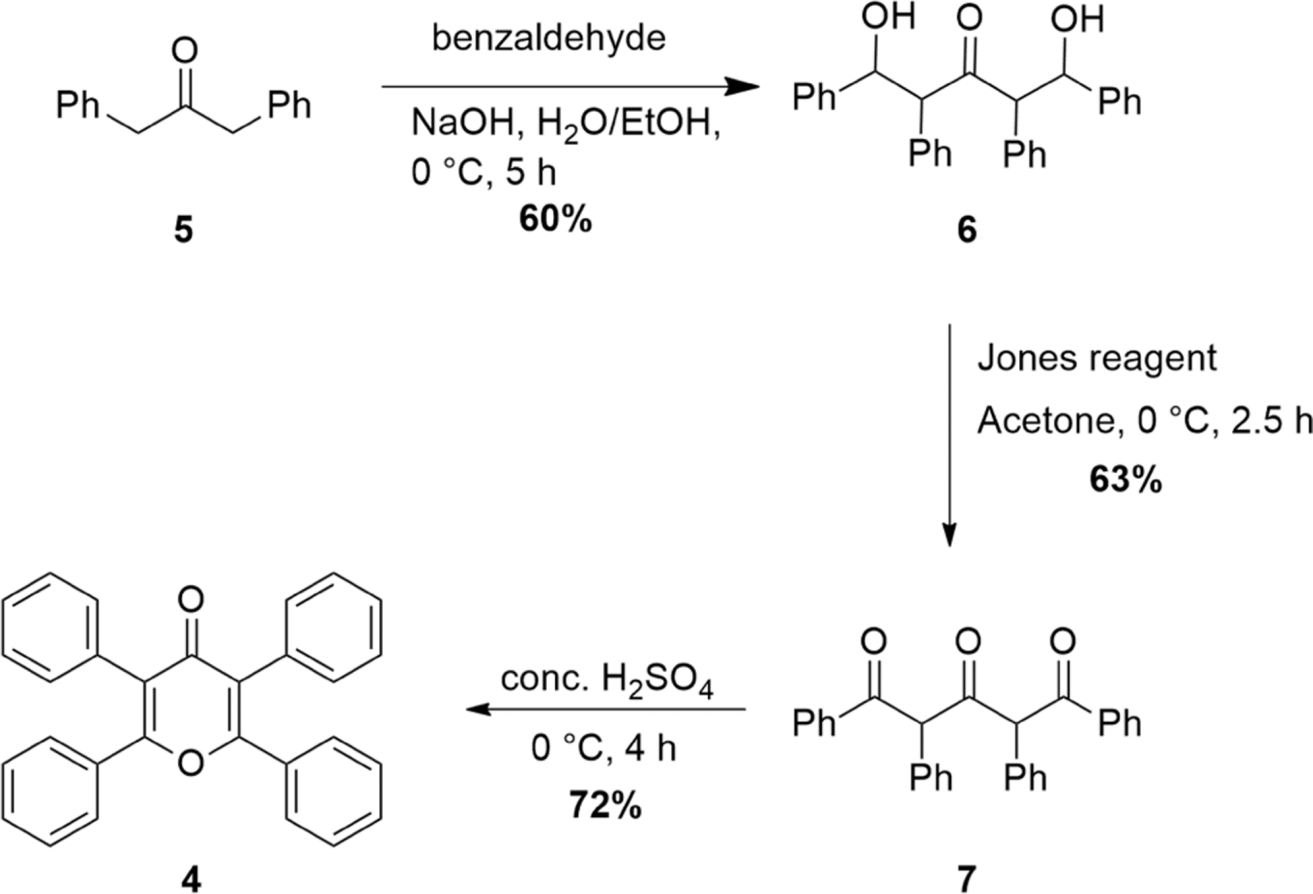
New Synthetic Route to Pyranone **4**

We planned an alternative pathway to synthesize pyranone **4**, which is illustrated in Scheme 1. The first step of the synthesis was modified from the study by Singh and Bhardwaj^12^ where they deprotonated deoxybenzoin by sodium hydroxide and performed aldol reaction with benzaldehyde in an ethanol/water (1:2) solvent system. 1,3-Diphenyl-2-propanone (**5**) was reacted with benzaldehyde in the presence of NaOH to afford diol **6**. The oxidation of diol **6** to **7** was tried with trichloroisocyanuric/TEMPO oxidation^13−15^ and oxidation with activated manganese dioxide,^16−18^ copper chloride,^19^ pyridinium dichromate (PDC), and pyridinium chlorochromate (PCC),^20^ but **7** could not be obtained. As a final trial, the Jones reagent was employed and **7** was synthesized successfully.^21^ At the final step, the cyclization of **7** to get pyranone **4** was successfully achieved in concentrated sulfuric acid.^22^ Molecules **6**, **7** and pyranone **4** were characterized by IR, ^1^H, ^13^C NMR, and high-resolution mass spectrometry (HRMS). Single crystal X-ray analysis of pyranone **4** was also conducted.

Although pyranone **4** is synthesized in three steps, the overall yield is 27%, which is higher than the reported yield in the literature. Moreover, derivatives of pyranone **4** can be synthesized with different ketones and benzaldehyde derivatives, which helps to widen the library of **4**-like molecules in the literature.

**Figure 6:**
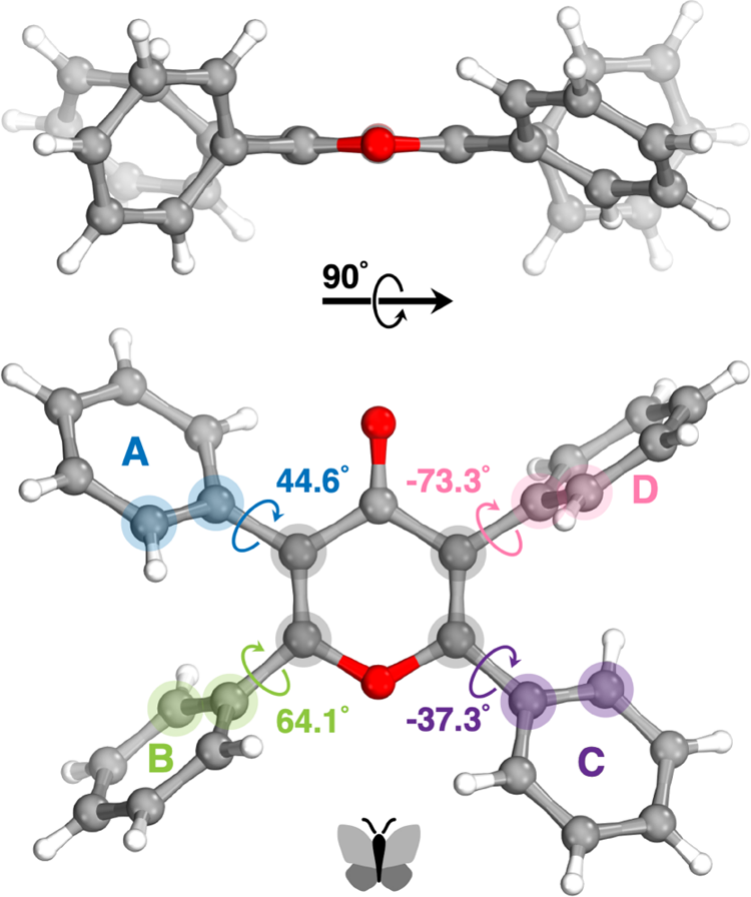
Crystal structure of pyranone **4** showing the dihedral angles of the phenyl rings.

As seen in Figure 6, the central core of pyranone **4** adopts a substantially planar conformation and the phenyl rings are oriented as butterfly wings. While the carbonyl functionality stays completely within the six-membered backbone, the phenyl rings (labeled as A, B, C, and D in Figure 6) are tilted with substantially different dihedral angles (i.e., 44.6°, 64.1°, −37.3°, and −73.3°, respectively). The C=C double bond lengths (1.337 and 1.345 Å) in the pyranone core are slightly different. These distances are notably longer than the double bond in the cyclohexene (1.326 Å). This implies that lone pairs of oxygen in the pyranone core take part in resonance. Further interpretation can be done by comparing the C=O double bond length in the pyranone core (1.268 Å) with a nonconjugated C=O double bond. Cyclohexanone C=O double bond length was reported as 1.225 Å, which is significantly shorter than the C=O double bond length in the pyranone core.^23,24^ The C–O bond distances on the pyranone ring are 1.381 and 1.361 Å. These differences can be explained by the better resonance of the oxygen lone pair to the phenyl ring that has a lower dihedral angle (ring A) than the pyranone ring.

**Figure 7:**
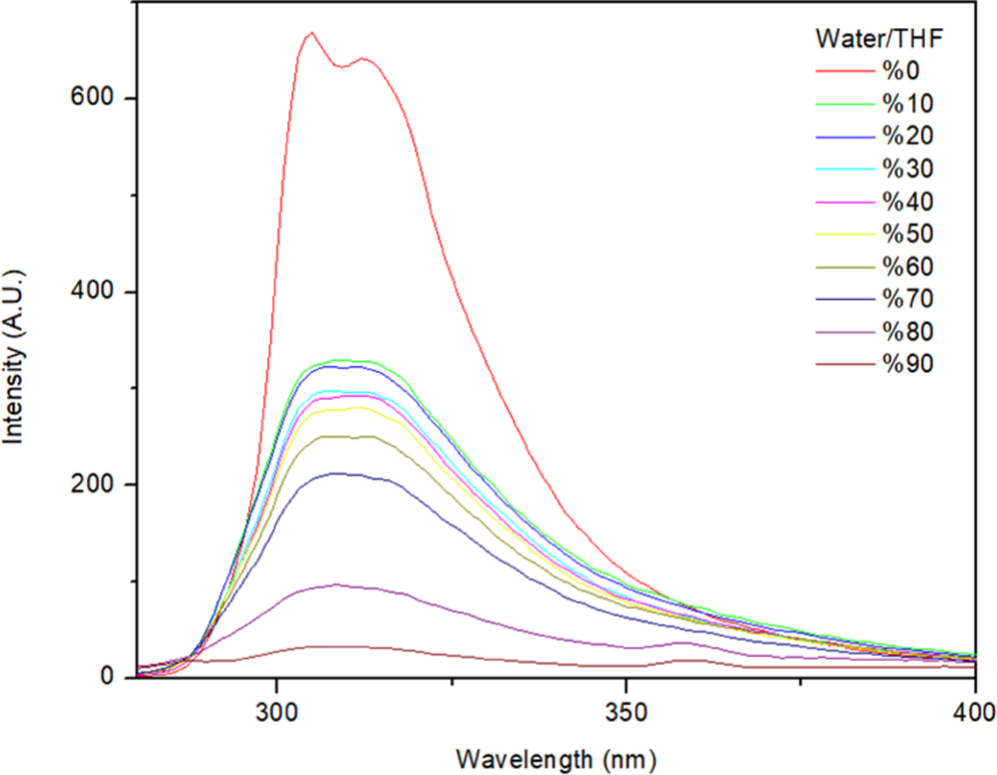
Fluorescence spectrum of pyranone **4** with varying water/THF.

We collected fluorescence spectra in a THF/H_2_O solvent system to determine whether pyranone **4** has ACQ or AIE properties (Figure 7). Attenuation of fluorescence was observed, as water percentage increased, which implies that pyranone **4** has the ACQ property instead of AIE, surprisingly. Also, the quantum yield of **4** was found to be 0.011, which was computed with respect to the tyrosine standard.

Solid-state fluorescence spectra of dioxin 1 and pyranone 4 were also measured (Figures S20 and S21), and they did not exhibit noticeable fluorescence in the solid state as expected.

We further conducted a theoretical study in an attempt to rationalize the opposite behavior observed for 2,3,5,6-tetraphenylpyrazine (TPP) and for 2,3,5,6-tetraphenyl-1,4-dioxin (TPD) and 2,3,4,5-tetraphenyl-4*H*-pyran-4-one (TPPO), i.e., AIE and ACQ, respectively. We calculated and analyzed the conformational preferences as well as the excited states of TPP, TPD, and TPPO via time-dependent density functional theory (TDDFT). For each molecule, two conformations of the phenyl rings were considered, further referred to as butterfly, where the orientation of the rings on one side is opposite to that of the rings on the other side, and propeller, where all four rings are oriented in the same direction. We found that for isolated molecules in implicit solvent (i.e., THF or water), the two conformations are nearly isoenergetic, with the propeller form slightly favored by a few tenth of kcal/mol. As previously mentioned elsewhere,^25^ the rotation of the rings is nearly barrier-free, and the molecules are expected to switch from one conformation to another rapidly in solution. The fact that only the butterfly conformation is observed in the crystals of all three molecules suggests that the propeller form does not allow efficient packing in the solid.

After extensive benchmarking of DFT functionals (see Table S17), we determined that these systems require range-separated functionals and selected ωB97X-D3. The basis set did not appear to have a significant effect on the results, which led us to move on with def2-SVP based on computational speed considerations. We further discuss results obtained with THF as an implicit solvent, and only notice that using water’s dielectric constant value instead did not affect the results of geometries nor the calculations of excited states significantly.

**Figure 8:**
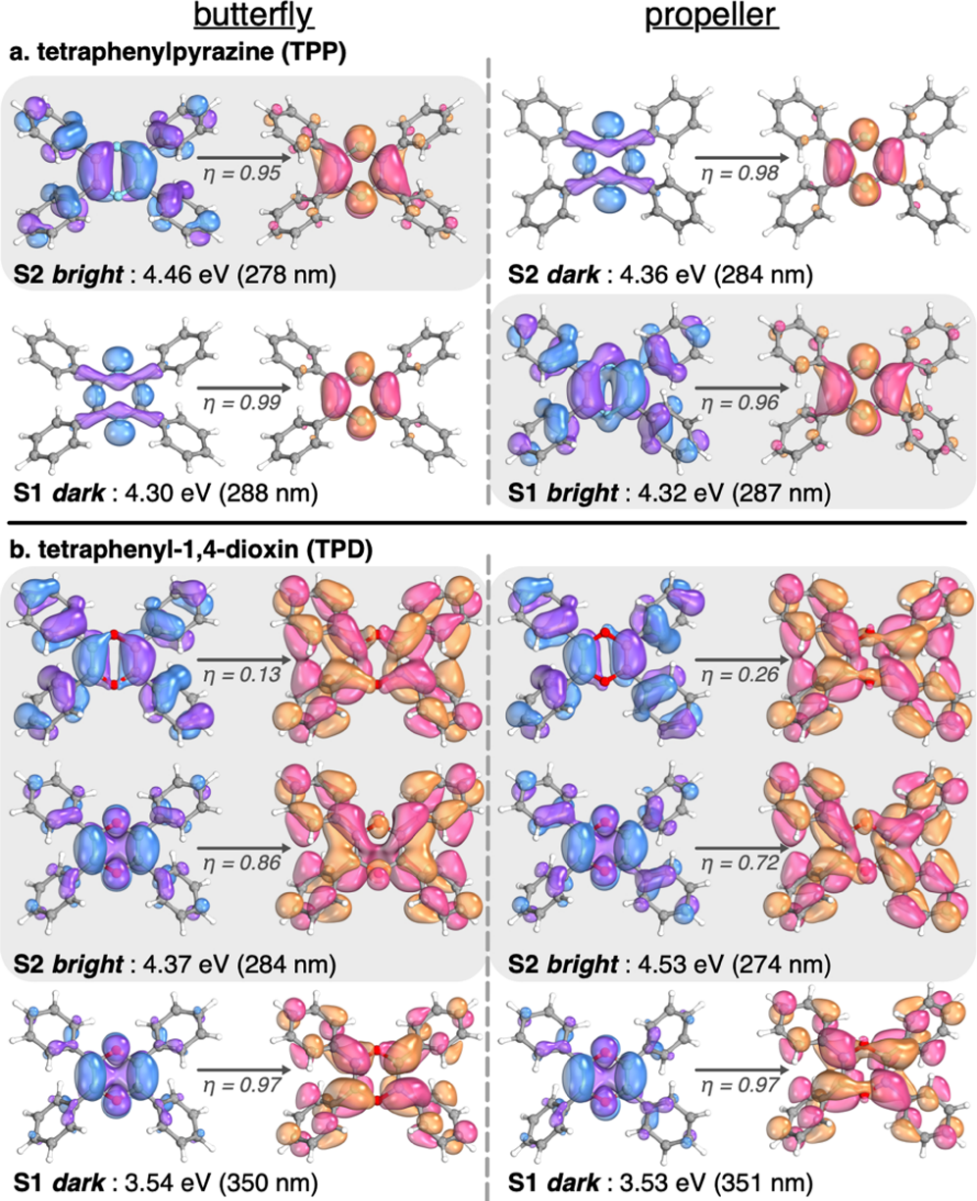
Natural transition orbitals for S1 and S2 states of TPP (a) and TPD (b) in implicit THF at the TD-ωB97X-D3/def2-SVP level. The occupation number (*η*) for the relevant NTO couples is given as well as the energy of the transition.

In Figure 8, we plotted relevant natural transition orbitals (NTOs) and the corresponding transition energy for few states of TPP and TPD in their ground-state geometries in butterfly and propeller conformations. Similar results are presented in Figure S24 for TPPO. The first two excited states of TPP (i.e., S1 and S2) are very close in energy in both butterfly and propeller conformations (i.e., 0.16 and 0.04 eV difference, respectively). We notice that the nature of the states does not change with the conformation, but their energy does, which results in an inversion of energy order. In the butterfly conformation, S1 is dark (i.e., cannot absorb or emit light), while S2 is bright (i.e., can absorb or emit light). In the propeller conformation, this situation is inverted. This indicates that the two states cross upon rotation of the phenyl rings, leading to quenching of fluorescence by internal conversion from the bright to dark state in solution, i.e., when the rotation is free. As the general consensus on the mechanism of AIE suggests, this switch from the bright to dark state is prohibited in the aggregate form since the rotation of the rings is hindered, letting the molecule fluoresce. After absorption, the molecular geometry relaxes in the excited state, thus lowering its energy. The relaxed energy of S1/S2 in butterfly and propeller conformations are 3.01/3.57 and 2.75/3.62 eV, respectively. It is noteworthy for the following discussion that the energy of the excited molecule does not match anymore with any energy level of its neighbors in their ground state when the aggregate is formed. The situation for TPD (and TPPO; see the SI) is fairly different. The separation between S1 and S2 is significantly larger than that in the case of TPP (i.e., 0.83 and 1.00 eV in butterfly and propeller conformations, respectively). More importantly, the order of the states does not change upon rotation of the rings, suggesting that internal conversion between the bright state (S2) and the dark state (S1) is unlikely. This is consistent with the observed fluorescence of TPD in solution.

**Figure 9:**
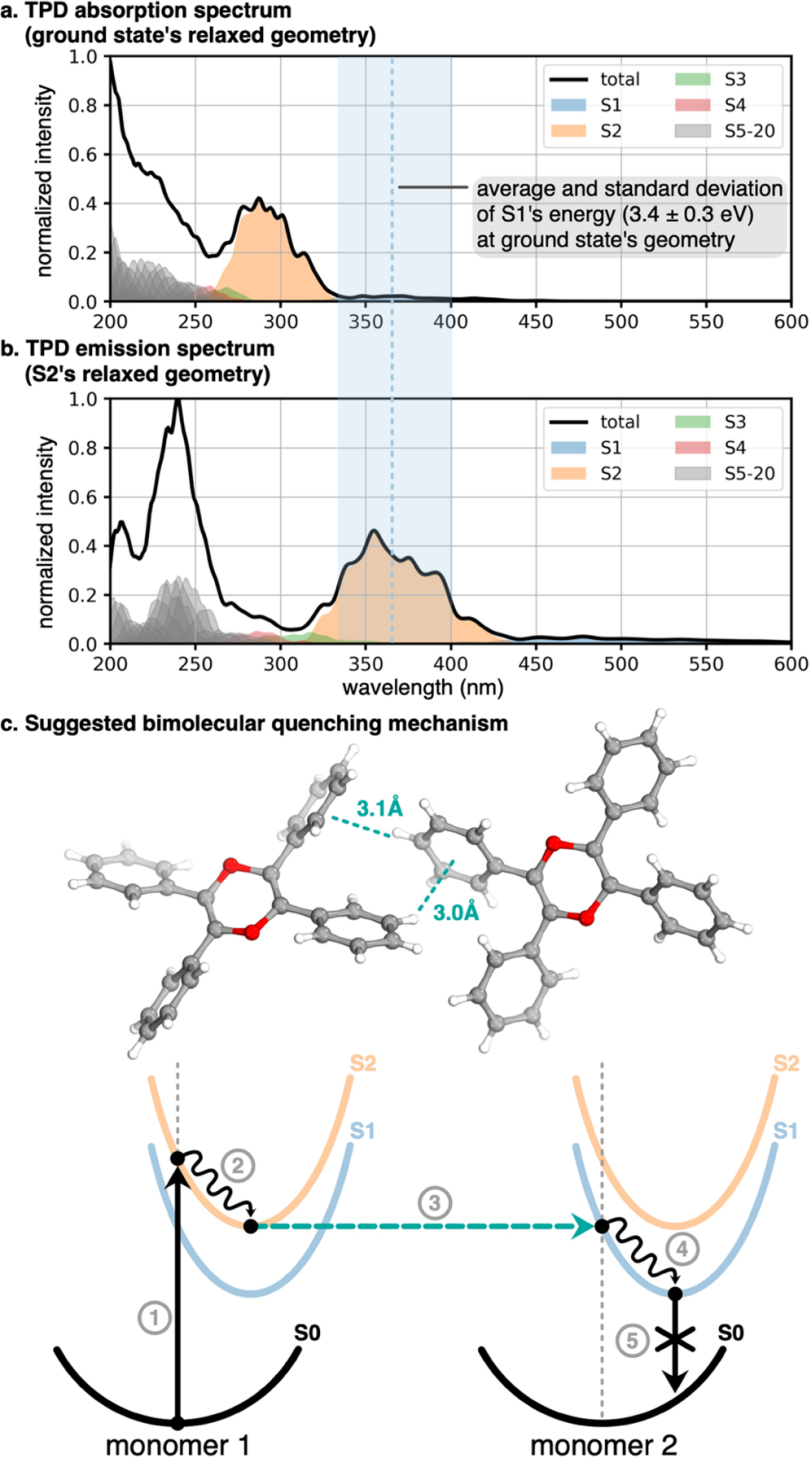
Absorption (a) and emission (b) spectra of TPD as obtained from a Wigner distribution of 400 geometries at 300 K. The average energy and corresponding standard deviation of the dark S1 state are shown in blue. A suggested bimolecular quenching mechanism is given in the bottom panel (c) with a representation of a dimer presenting a T-shaped intermolecular interaction between phenyl rings, as extracted from the crystal structure.

We further calculated the absorption and emission spectra of TPD (in the ground state and S2 relaxed geometries, respectively) from a Wigner distribution of conformations around the minima of butterfly and propeller conformations at 300 K (i.e., 200 geometries each). The spectra are presented in Figure 9a,b. The wide S2 absorption band centered around 280 nm is red-shifted in the emission spectrum and peaks at about 360 nm. We notice that the S2 band in the S2 relaxed geometry overlaps significantly with the energy distribution of the dark S1 state in the ground-state geometry. This lets us suggest a plausible quenching mechanism that we schematically represent in Figure 9c, which most likely also applies to TPPO after inspection of its electronic structure in Figure S24.

Our suggested mechanism involves two molecules forming a dimer, or two neighbors in a larger aggregate, further referred to as monomer 1 and monomer 2. At first, monomers 1 and 2 are in their ground electronic states and corresponding geometries when monomer 1 absorbs a photon via vertical excitation to its bright S2 state (1). Monomer 1 further relaxes to its S2 minimum geometry, which our calculations showed to match the energy of S1 of monomer 2 in its ground-state geometry (2). Inspection of the crystal structure of TPD shows interactions between phenyl rings of neighboring monomers, forming T-shaped complexes with hydrogen to ring center distances of about 3 Å. This close contact and the energy match between the states of excited monomer 1 and ground state monomer 2 lets us speculate an intermolecular internal conversion (3) leading to monomer 1 returning to its ground state in a nonradiative manner and monomer 2 reaching its excited state S1. Monomer 2 further relaxes to S1 geometry (4), and since S1 is dark, no fluorescence can be observed (5). The energy must then dissipate by other nonradiative means. A similar idea was suggested by Zhang et al., with an S1 → S0 internal conversion promoted by intermolecular hydrogen bonding upon aggregation.^26^

Rather than closing the story on the mechanism of ACQ, our intention is to open new doors for future investigations of this puzzling phenomenon. The next step in this direction is to conduct calculations on aggregates via molecular dynamics in the framework of nonadiabatic simulations.^27,28^ We anticipate that such models should include a large number of molecules, the identification of an ensemble of relevant aggregate forms, and the calculation of a large number of nonadiabatic molecular dynamics simulations at a fairly high level of quantum chemistry to probe the dynamics of the electronic structure and its relaxation routes. While such a study would be computationally intensive, we trust that our results will help as a guide and constitute a valuable starting point.

## Conclusions

2,3,5,6-Tetraphenyl-1,4-dioxin **1** (TPD) and 2,3,4,5-tetraphenyl-4*H*-pyran-4-one **4** (TPPO) were synthesized and investigated for their optical properties upon aggregation. We introduced a new, modular synthesis pathway of pyranone **4** that enabled us to synthesize its derivatives using substituted aromatic starting materials. It was observed that dioxin **1** is slightly distorted from planarity, while pyranone **4** has a planar core and four phenyl rings with butterfly or propeller geometry. The butterfly conformation appears to be favored in the aggregate form. Despite having a similar structure to known molecules with aggregation-induced emission (AIE) properties (e.g., 2,3,5,6-tetraphenylpyrazine, TPP), both dioxin **1** and pyranone **4** showed the aggregation-caused quenching (ACQ) property instead.

These results are perplexing since satisfactory explanation based on the RIM mechanism which is proposed in the literature is not possible. We shed light on this puzzling phenomenon via TDDFT calculations that led us to suggest a bimolecular quenching mechanism involving intermolecular, nonradiative internal conversion. The chain of thoughts that led us to this hypothesis is based on electronic structure differences observed between two molecules, i.e., TPP (AIE property) and TPD (ACQ property), and can be summarized as follows. In solution, TPP and TPD can adopt two main conformations, referred to as butterfly and propeller, connected by a nearly barrier-free rotation of their phenyl rings. In its butterfly conformation, TPP’s S1 and S2 states are close in energy and, respectively, dark and bright. Switching to a propeller conformation inverts the energy order of these two states, suggesting an efficient internal conversion that explains the lack of fluorescence in solution. In an aggregate, the conformation of the phenyl rings is thought to be locked, preventing internal conversion and avoiding quenching of fluorescence resulting in an AIE phenomenon for TPP. In TPD, however, the low-lying excited states are well separated in energy and the rotation of the phenyl rings does not lead to any inversion of energy levels. Internal conversion is, therefore, unlikely, which is compatible with the experimentally observed fluorescence of TPD in solution.

The ACQ mechanism is seldom discussed in the literature. Notably, Zhang et al. suggested an S1 → S0 internal conversion induced by intermolecular hydrogen bonding in the aggregate form of a near-infrared dye.^26^ Our calculations of dynamical absorption and emission spectra of TPD show that the energy of the relaxed S2 state of TPD significantly overlaps with the energy of the S1 level in the ground-state geometry. This result lets us speculate that a molecule of TPD excited to its S2 state will further transfer energy in a nonradiative manner to a neighboring molecule via S2 → S1 intermolecular internal conversion. The S1 state of TPD being dark, the final relaxation step must be nonradiative as well, leading to an effective quenching of fluorescence in the aggregate form. We trust that our results will trigger more elaborate theoretical studies on the subject of ACQ, potentially via large-scale nonadiabatic dynamics methods, to validate or challenge our suggested mechanism.

## Supporting Information Available

Additional experimental details, materials and methods including IR, ^1^H, ^13^C NMR, HRMS, and single crystal X-ray analysis results, and additional theoretical data (PDF)

## Abbreviations

ACQ: aggregation-caused quenching
AIE: aggregation-induced emission
CSD: Cambridge structural database
HRMS: high-resolution mass spectrometry
RIM: restriction of intramolecular motion
RIR: restriction of intramolecular rotation
RIV: restriction of intramolecular vibration
TEMPO: (2,2,6,6-tetramethylpiperidin-1-yl)oxyl
TPD: 2,3,5,6-Tetraphenyl-1,4-dioxin
TPP: 2,3,5,6-tetraphenylpyrazine
TPPO: 2,3,4,5-tetraphenyl-4*H*-pyran-4-one
TsOH: *p*-toluenesulfonic acid
XRD: X-ray diffraction
