# Supplementary material for: Tetraphenyl-1,4-dioxin and Tetraphenyl-pyrane-4-one: Old Molecules, New Insights
Source: ACS Omega. 2023 May 22;8(22):19656–62. doi: 10.1021/acsomega.3c01226 (PMC10249104; doi:10.1021/acsomega.3c01226)
Supplement: Supplementary file 1 — ao3c01226_si_001.pdf [file ao3c01226_si_001.pdf]

Supplementary Information

Tetraphenyl-1,4-dioxin and Tetraphenyl-pyrane-4-one: Old Molecules, New Insights

Medine Soydan[1], Burcu Okyar[1], Yunus Zorlu[2], Antoine Marion[1], Salih Özçubukçu[1,*]
1. Department of Chemistry, Middle East Technical University, 06800, Ankara, Turkey
2. Department of Chemistry, Gebze Technical University, 41400, Kocaeli, Turkey
*Corresponding Author

1.1 List of Figures 2
1.2 List of Tables 3
2 SUPPLEMENTARY INFORMATION 4
2.1 The Synthesis of molecules 4
2.1.1 The synthesis of 2,3,5,6-tetraphenyl-1,4-dioxin (1) 4
2.1.2 The synthesis of 1,5-dihydroxy-1,2,4,5-tetraphenylpenta-3-one (6) 5
2.1.3 The synthesis of 1,2,4,5-tetraphenylpentane-1,3,5-trione (7) 5
2.1.4 Synthesis of 2,3,5,6-tetraphenyl-4H-pyran-4-one (4) 5
2.2 ^{1}H AND ^{13}C NMR SPECTRUM 6
2.3 MASS SPECTRUM 9
2.4 IR SPECTRUM 11
2.5 Fluorescence Spectrum 13
2.5.1 Fluorescence spectrum of 2,3,5,6-tetraphenyl-1,4-dioxin (1) 13
2.5.2 Fluorescence spectrum of 2,3,4,5-tetraphenyl-4H-pyran-4-one (4) 13
2.6 Quantum Yield Calculation for Dioxin 1 14
2.7 Quantum Yield Calculation for 2,3,4,5-tetraphenyl-4H-pyran-4-one (4) 15
2.8 Solid Fluorescence Analysis 16
2.8.1 Solid fluorescence spectrum of dioxin 1 16
2.8.2 Solid fluorescence of pyranone 4 17
2.9 XRD ANALYSIS 18
2.9.1 XRD data of 2,3,5,6-tetraphenyl-1,4-dioxin (Dioxin 1) 18
2.9.2 XRD data of 2,3,4,5-tetraphenyl-4H-pyran-4-one (4) 22
2.10 Theoretical studies 28
2.11 REFERENCES 29

1.1 List of Figures

Figure S1. ^{1}H NMR spectrum of 2,3,5,6-tetraphenyl-1,4-dioxin (**1**) in $CDCl_3$. 6
Figure S2.^{13}C NMR spectrum of 2,3,5,6-tetraphenyl-1,4-dioxin (**1**) in $CDCl_3$. 6
Figure S3. ^{1}H NMR spectrum of 1,5-dihydroxy-1,2,4,5-tetraphenylpenta-3-one (**6**) in DMSO-d_6. 7
Figure S4.^{13}C NMR spectrum of 1,5-dihydroxy-1,2,4,5-tetraphenylpenta-3-one (**6**) in DMSO-d_6. 7
Figure S5. ^{1}H NMR spectrum of 1,2,4,5-tetraphenylpentane-1,3,5-trione (**7**) in $CDCl_3$. 8
Figure S6. ^{13}C NMR of spectrum 1,2,4,5-tetraphenylpentane-1,3,5-trione (**7**) in $CDCl_3$. 8
Figure S7. ^{1}H NMR spectrum of 2,3,5,6-tetraphenyl-4H-pyran-4-one (**4**) in $CDCl_3$. 9
Figure S8. ^{13}C NMR spectrum of 2,3,5,6-tetraphenyl-4H-pyran-4-one (**4**) in $CDCl_3$. 9
Figure S9. HRMS spectrum of 2,3,5,6-tetraphenyl-1,4-dioxin (**1**). 10
Figure S10. HRMS spectrum of 1, 5-dihydroxy-1,2,4,5-tetraphenylpenta-3-one (**6**). 10
Figure S11. HRMS spectrum of 1,2,4,5-tetraphenylpentane-1,3,5-trione (**7**). 10
Figure S12. HRMS spectrum of 2,3,5,6-tetraphenyl-4H-pyran-4-one (**4**) 11
Figure S13. FT-IR spectrum of 1,5-dihydroxy-1,2,4,5-tetraphenylpenta-3-one (**6**). 11
Figure S14. FT-IR spectrum of 1,2,4,5-tetraphenylpentane-1,3,5-trione (**7**). 12
Figure S15. FT-IR spectrum of 2,3,5,6-tetraphenyl-4H-pyran-4-one (**4**). 12
Figure S16. The fluorescence spectrum of 2,3,5,6-tetraphenyl dioxin (dioxin **1**) in THF/H_2O mixture. 13
Figure S17. Fluorescence spectrum of 2,3,4,5-tetraphenyl-4H-pyran-4-one (**4**) with varying water %. 14
Figure S18. Integrated fluorescence spectrum area versus absorbance graph for dioxin **1**. 15
Figure S19. Integrated fluorescence spectrum area versus absorbance graph for 2,3,4,5-tetraphenyl-4H-pyran-4-one (**4**)......16
Figure S20. Solid fluorescence spectrum of dioxin **1** with 275 nm excitation wavelength. 17
Figure S21. Solid fluorescence spectrum of pyranone **4** with 260 nm excitation wavelength. 18
Figure S22. The crystal structure of dioxin **1**, showing the atom numbering scheme. 19
Figure S23. The crystal structure of pyranone **4**, showing the atom numbering scheme. 23
Figure S24. Natural transition orbitals for S1, S2, and S3 states of TPPO in implicit THF at the TD-wB97X-D3/def2-SVP level. The occupation number (η) for the relevant NTO couples is given as well as the energy of the transition. 29

1.2 List of Tables

Table S1. The mixtures of 2,3,4,5-tetraphenyl-4H-pyran-4-one (4) in water/THF system 13

Table S2. Crystal data and structure refinement for dioxin **1.** 19

Table S3. Fractional Atomic Coordinates ($\times 10^4$) and Equivalent Isotropic Displacement Parameters ($Å^2\times 10^3$) for dioxin **1**. U_{eq} is defined as 1/3 of of the trace of the orthogonalised U_{IJ} tensor. 20

Table S4. Anisotropic Displacement Parameters ($Å^2\times 10^3$) for dioxin **1**. The Anisotropic displacement factor exponent takes the form: $-2\pi^2[h^2a^{*2}U_{11}+2hka^*b^*U_{12}+\ldots]$. 20

Table S5. Bond Lengths for dioxin **1**. 20

Table S6. Bond Angles for dioxin **1**. 21

Table S7. Torsion Angles for dioxin **1**. 21

Table S8. Hydrogen Atom Coordinates ($Å\times 10^4$) and Isotropic Displacement Parameters ($Å^2\times 10^3$) for dioxin **1**. 21

Table S9. Atomic Occupancy for dioxin **1**. 22

Table S10. Crystal data and structure refinement for 2,3,4,5-tetraphenyl-4H-pyran-4-one (**4**). 23

Table S11. Fractional Atomic Coordinates ($\times 10^4$) and Equivalent Isotropic Displacement Parameters ($Å^2\times 10^3$) for pyranone **4**. U_{eq} is defined as 1/3 of of the trace of the orthogonalised U_{IJ} tensor. 23

Table S12. Anisotropic Displacement Parameters ($Å^2\times 10^3$) for pyranone **4**. The Anisotropic displacement factor exponent takes the form: $-2\pi^2[h^2a^{*2}U_{11}+2hka^*b^*U_{12}+\ldots]$. 24

Table S13. Bond Lengths for pyranone **4**. 25

Table S14. Bond Angles for pyranone **4**. 25

Table S15. Torsion Angles for pyranone **4.** 26

Table S16. Hydrogen Atom Coordinates ($Å\times 10^4$) and Isotropic Displacement Parameters ($Å^2\times 10^3$) for pyranone **4.** 27

Table S17. Benchmark of density functionals and basis sets for the first six singlet states of TPD. The energy of the transition is given in electron volt and the oscillator strength is in parenthesis. According to the experimental absorption spectrum of TPP in THF, there should be no absorption above 300-350 nm (i.e., not bright state is expected bellow 3.5 eV). Only M06-2X, and the two range-separated functionals CAM-B3LYP, and wB97X-D3 seem valid for this system. The others tend to over-stabilize some dark and/or bright states, although there is, admittedly, no experimental support for the position of dark states. 28

SUPPLEMENTARY INFORMATION

Materials

Commonly used solvents such as DCM, ACN, THF, Et_2O, DMF, ethanol and methanol were obtained from Merck. Solvents such as EtOAc, hexane and DCM employed to perform column chromatography, extraction or washing were technical grade and they were dried with calcium chloride in distillation system prior to usage. Starting materials were obtained from commercial sources and they were employed without further purification. Deuterated solvents were also provided by Merck. Column chromatography purifications were performed with Merck Silica gel 60 (0.063-0.20 mm). Moreover, the progress of the reactions was traced with TLC plates which were purchased from Merck. TLC plates were coated with silica gel and contain F254 fluorescent indicator which enables the visualization at 254 nm. Deionized water was obtained in Millipore Simplicity 185 water purification system and utilized in further applications.

Instrumentation

Characterization or analysis of the synthesized molecules were performed via various instruments. The Nuclear Magnetic Spectrum of molecules obtained on Bruker Spectrospin Advance DPX 400 spectrometer. Chemical shifts were presented in parts per million (ppm) and TMS was utilized as internal standard. HRMS (ESI-TOF-MS) data were collected with Waters SYNAPT G1 MS system in positive mode, 50-1000 Da interval. FT-IR measurements were done in Thermo Scientific Nicolet is10 instrument. UV spectrum was collected via Agilent Technologies Cary 8454 UV-Vis spectrometer. Fluorescence spectra were collected by using Agilent Technologies Cary Eclipse Fluorescence Spectrophotometer. XRD data was collected through Bruker APEX II QUAZAR three-circle diffractometer at 298 K.

2.1 The Synthesis of molecules

2.1.1 The synthesis of 2,3,5,6-tetraphenyl-1,4-dioxin (1)

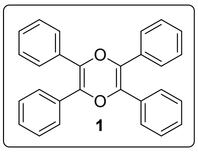

1

Benzoin (18.5 g, 87.2 mmol) was dissolved in 140 mL methanol. Dry HCl passed through the solution for 8 hours. The reaction was bubbled for 8 hours, addition to this duration, it was stirred for 48 hours at room temperature. The solution was filtered via Büchner funnel and dried overnight. 8.3 g white powder was collected as a mixture of 2-methoxy-2,3,5,6-tetraphenyl-2,3-dihydro-1,4-dioxin and 2,5-dimethoxy-2,3,5,6-tetraphenyl-1,4-dioxane.Then, all the collected product mixture (8.3 g) white precipitate was reacted with *p*-TsOH (1.57 g, 9.12 mmol) in 27 mL dry acetic anhydride at 160 °C for 5 minutes. The reaction was cooled at room temperature and put in 0 °C ice-bath for an hour. Yellow precipitate was formed and filtered through filter paper. Then, the precipitate was washed with cold acetic anhydride and dried over freeze dryer. 6.08 g bright yellow precipitate of **1** was gathered. Yield was computed as 18%.

^{1}H NMR (400 MHz, $CDCl_3$) δ 7.34 – 7.27 (m, 2H), 7.19 – 7.13 (m, 3H).

^{13}C NMR (100 MHz, $CDCl_3$) δ 136.0, 132.9, 128.3, 128.2, 128.1.

HRMS $C_{28}H_{20}O_2$ [M^+]: Calculated 388.1463, found 388.1481.

2.1.2 The synthesis of 1,5-dihydroxy-1,2,4,5-tetraphenylpenta-3-one (6)

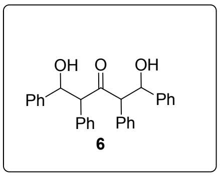

6

NaOH (8 equivalent, 38.0 mmol, and 1.52 g) in total of 7.5 mL of ethanol-water(1:2) was cooled in ice bath. Then, 1,3-diphenylacetone (1.00 g, 4.75 mmol) was added and stirred for 3 minutes, then benzaldehyde (5 equivalent, 23.8 mmol) was introduced. The reaction mixture was stirred for 5 h in ice bath. Then, the product was filtered through filter paper and washed with excess, cold DCM. 1.16 g white colored product was obtained. Yield was computed to be 60%.[1]

^{1}H NMR (400 MHz, DMSO) δ 7.11 (m, 10H), 7.07 – 6.85 (m, 6H), 6.74 (m, 4H), 5.56 (d, *J* = 1.7 Hz, 2H), 5.22 (d, *J* = 6.7 Hz, 2H), 4.34 (d, *J* = 9.2 Hz, 2H).

^{13}C NMR (100 MHz, DMSO) δ 209.1, 143.2, 135.7, 129.5, 128.0, 127.8, 127.5, 127.2, 126.8, 75.6, 66.9.
HRMS $C_{29}H_{26}O_3$ $[M+Na]^+$: Calculated 445.1780, found 445.1781.

2.1.3 The synthesis of 1,2,4,5-tetraphenylpentane-1,3,5-trione (7)

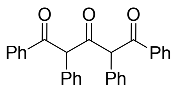

7

2.67 M Jones reagent was prepared as follows. 2.67 g CrO_3 was dissolved in 3.0 mL water, 2.3 mL concentrated H_2SO_4 was added to this solution and the solution was diluted to 10 mL to obtain 2.67 M Jones reagent. 1,5-dihydroxy-1,2,4,5-tetraphenylpentan-3-one (**6**) (200 mg, 0.473 mmol) was dissolved in 5 mL acetone and cooled to 0 °C in ice bath. Freshly prepared Jones reagent (2.88 equivalent) was added dropwise in 30 minutes. Reaction was stirred for additional 2 h at 0 °C. At the end of the reaction, 10 mL of isopropyl alcohol was added to deactivate the rest of Jones reagent. Then, diethyl ether extraction was performed twice, then organic fractions were collected and washed with distilled water and saturated $NaHSO_4$, respectively. Organic phase was dried over Na_2SO_4 and evaporated with rotary evaporator. The product was recrystallized with EtOH : DCM (90:10) solvent system. 114.5 mg white colored **7** was obtained. The yield was calculated as 63% yield.[2]

^{1}H NMR (400 MHz, $CDCl_3$) δ 7.88-7.81 (m, 4H), 7.56-7.49 (m, 2H), 7.42-7.35 (m, 5H), 7.33-7.24 (m, 9H), 6.02 (s, 2H).
^{13}C NMR (101 MHz, $CDCl_3$) δ 199.5, 194.7, 133.4, 132.4, 129.9, 128.9, 128.7, 128.5, 128.3, 128.1, 65.8.
HRMS $C_{29}H_{23}O_3$ $[M+H]^+$: Calculated 419.1647, found 419.1696.

2.1.4 Synthesis of 2,3,5,6-tetraphenyl-4H-pyran-4-one (4)

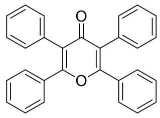

4

1,2,4,5-tetraphenylpentane-1,3,5-trione(**7**) (80.0 mg, 0.190 mmol) was added to a reaction vessel and put into ice bath. Ice cooled concentrated H_2SO_4 (300 µL) was added. Reaction mixture was stirred for 4 h at 0 °C. After 4 h, ice cooled water (3.5 mL) was added and then saturated $NaHCO_3$ was added slowly to neutralize H_2SO_4. Then, extraction with DCM was performed, organic fractions were collected, dried with Na_2SO_4 and DCM was removed under vacuum. The product was recrystallized with EtOH: DCM (90:10) solvent system. 55.7 mg white colored **4** was obtained and the yield was found as 72% yield.[3]

^{1}H NMR (400 MHz, $CDCl_3$) δ 7.45 – 7.40 (m, 4H), 7.40 – 7.34 (m, 4H), 7.31 (d, J = 5.1 Hz, 12H).
^{13}C NMR (101 MHz, $CDCl_3$) δ 177.7, 160.5, 132.7, 132.4, 131.0, 129.9, 129.3, 128.1, 127.6, 125.7.
HRMS $C_{29}H_{21}O_2$ $[M+H]^+$: Calculated 401.1542, found 401.1543.

2.2 ^{1}H AND ^{13}C NMR SPECTRUM

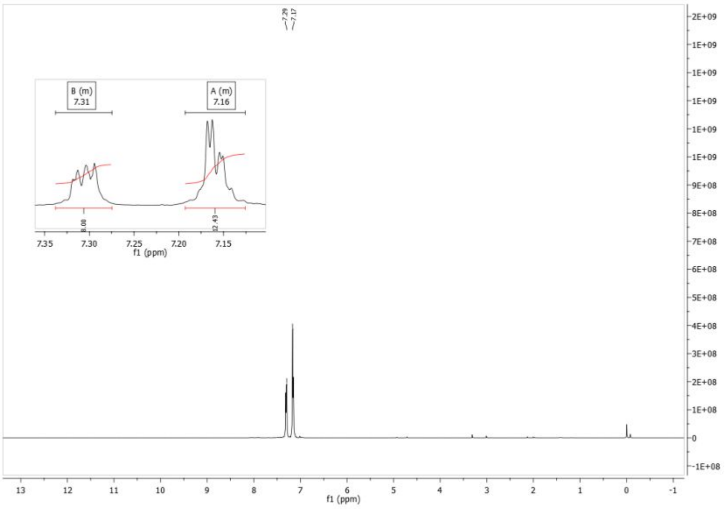

Figure S1. ^{1}H NMR spectrum of 2,3,5,6-tetraphenyl-1,4-dioxin (**1**) in $CDCl_3$.

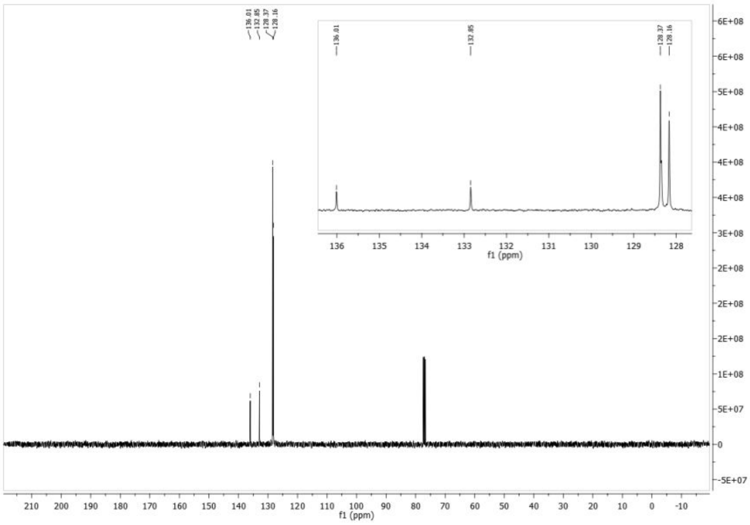

Figure S2. ^{13}C NMR spectrum of 2,3,5,6-tetraphenyl-1,4-dioxin (**1**) in $CDCl_3$.

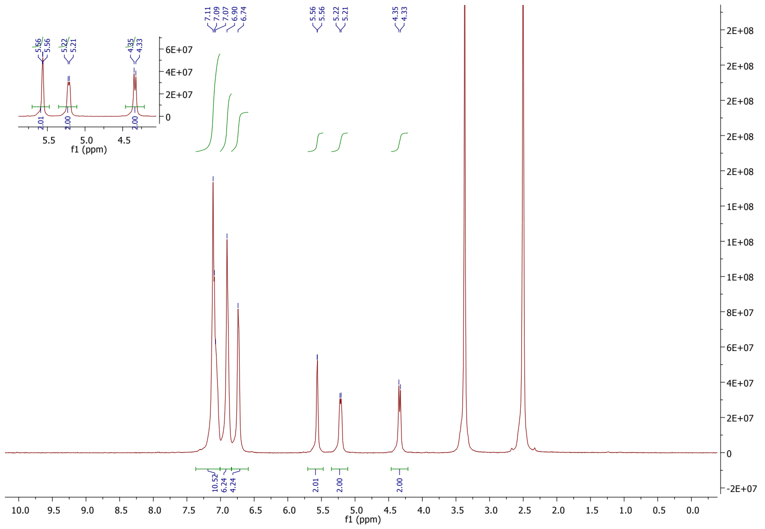

Figure S3. ^{1}H NMR spectrum of 1,5-dihydroxy-1,2,4,5-tetraphenylpenta-3-one (**6**) in DMSO-d_6.

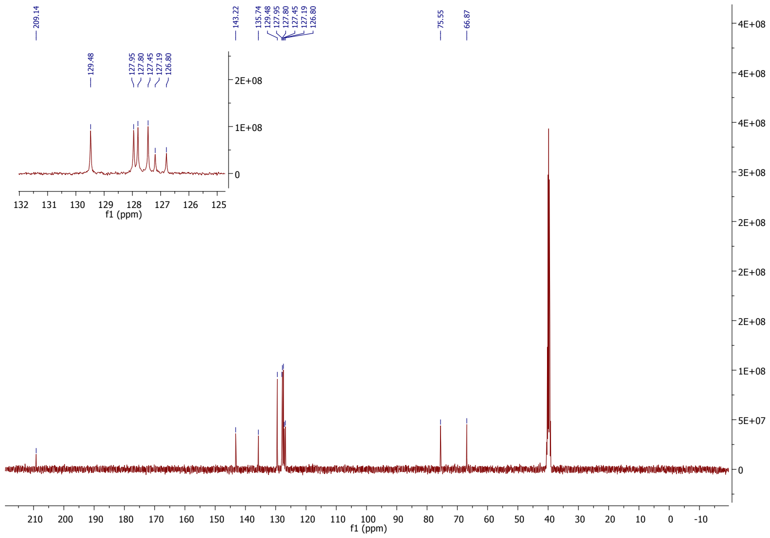

Figure S4.^{13}C NMR spectrum of 1,5-dihydroxy-1,2,4,5-tetraphenylpenta-3-one (**6**) in DMSO-d_6.

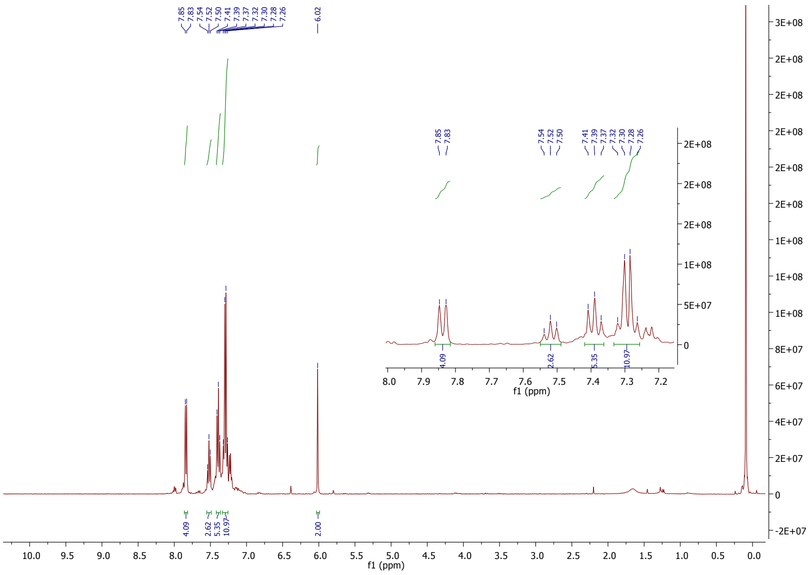

Figure S5. ^{1}H NMR spectrum of 1,2,4,5-tetraphenylpentane-1,3,5-trione (**7**) in $CDCl_3$.

.

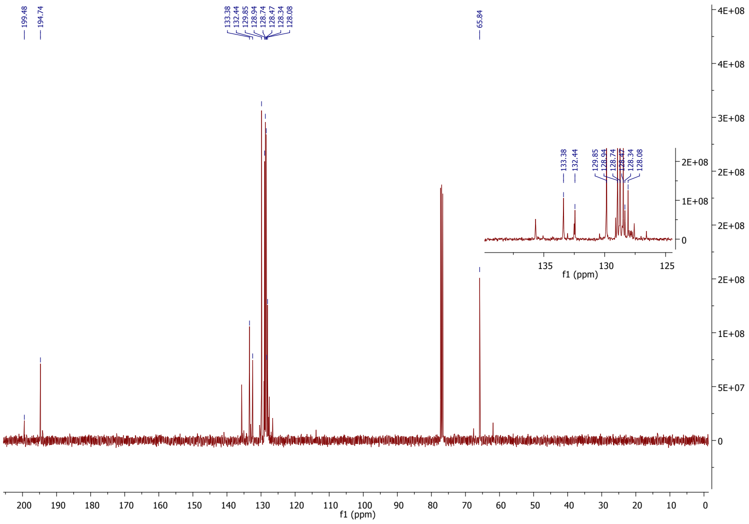

Figure S6. ^{13}C NMR of spectrum 1,2,4,5-tetraphenylpentane-1,3,5-trione (**7**) in $CDCl_3$.

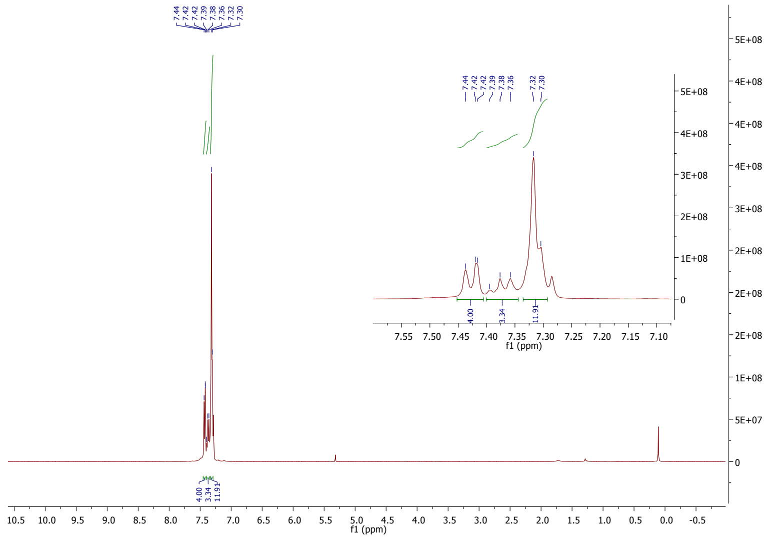

Figure S7. ^{1}H NMR spectrum of 2,3,5,6-tetraphenyl-4H-pyran-4-one (**4**) in $CDCl_3$.

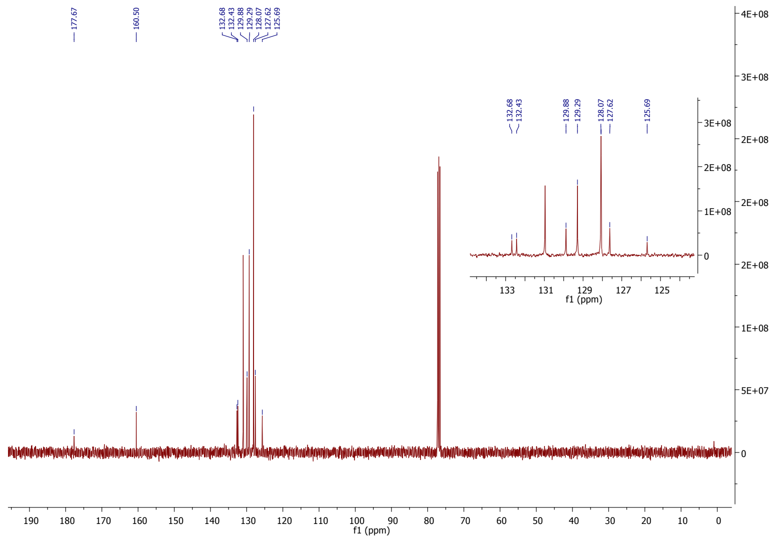

Figure S8. ^{13}C NMR spectrum of 2,3,5,6-tetraphenyl-4H-pyran-4-one (**4**) in $CDCl_3$.

2.3 MASS SPECTRUM

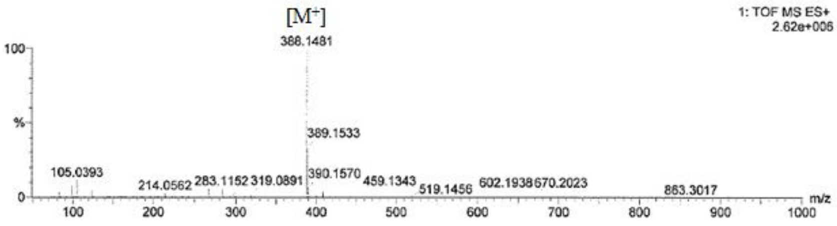

Figure S9. HRMS spectrum of 2,3,5,6-tetraphenyl-1,4-dioxin (**1**).

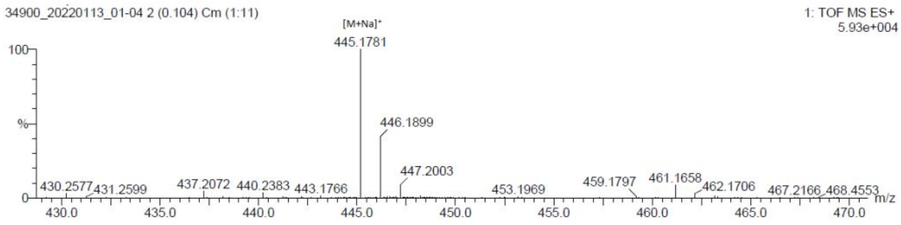

Minimum:				-5.5			
Maximum:		1000.0	1000.0	1000.0			
Mass	Calc. Mass	mDa	PPM	DBE	i-FIT	i-FIT (Norm)	Formula
445.1781	445.1780	0.1	0.2	16.5	1151.6	0.0	C29 H26 O3 Na

Figure S10. HRMS spectrum of 1, 5-dihydroxy-1,2,4,5-tetraphenylpenta-3-one (**6**).

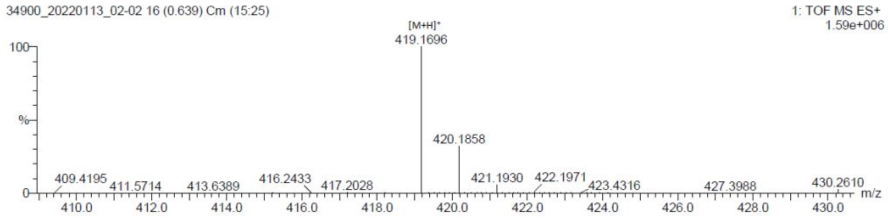

Minimum:				-5.5			
Maximum:		1000.0	1000.0	1000.0			
Mass	Calc. Mass	mDa	PPM	DBE	i-FIT	i-FIT (Norm)	Formula
419.1696	419.1647	4.9	11.7	18.5	1333.4	0.0	C29 H23 O3

Figure S11. HRMS spectrum of 1,2,4,5-tetraphenylpentane-1,3,5-trione (**7**).

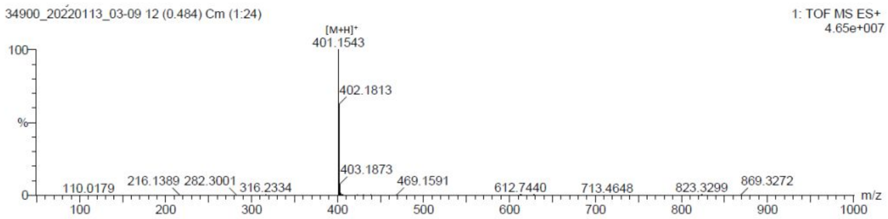

Minimum:				-5.5			
Maximum:		1000.0	1000.0	1000.0			
Mass	Calc. Mass	mDa	PPM	DBE	i-FIT	i-FIT (Norm)	Formula
401.1543	401.1542	0.1	0.2	19.5	2211.2	0.0	C29 H21 O2

Figure S12. HRMS spectrum of 2,3,5,6-tetraphenyl-4H-pyran-4-one (**4**) .

2.4 IR SPECTRUM

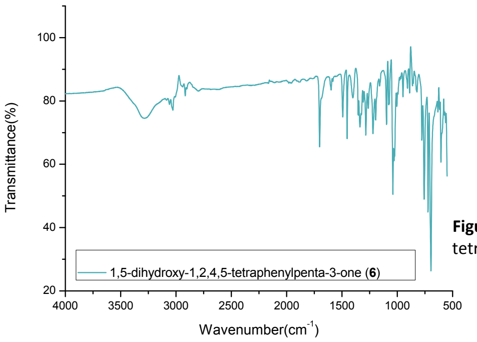

Figure S13. FT-IR spectrum of 1,5-dihydroxy-1,2,4,5-tetraphenylpenta-3-one (**6**).

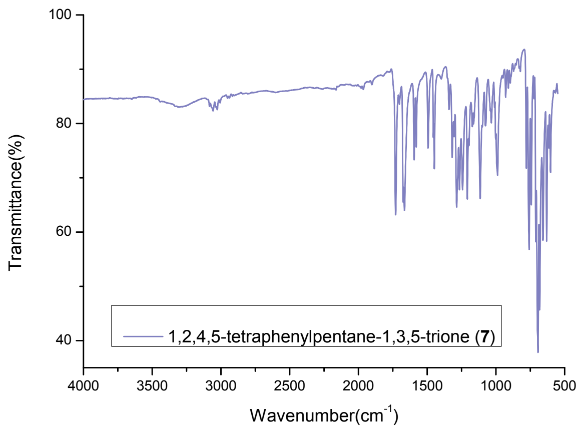

Figure S14. FT-IR spectrum of 1,2,4,5-tetraphenylpentane-1,3,5-trione (**7**).

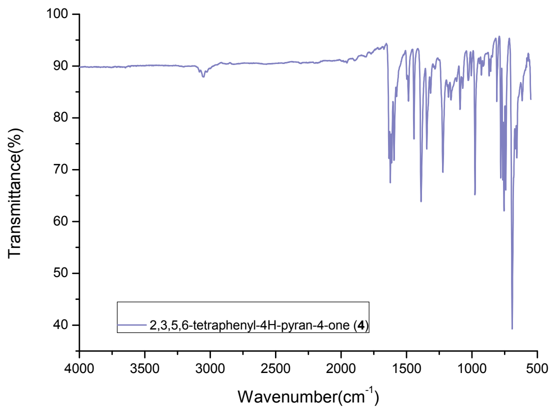

Figure S15. FT-IR spectrum of 2,3,5,6-tetraphenyl-4H-pyran-4-one (**4**).

2.5 Fluorescence Spectrum

2.5.1 Fluorescence spectrum of 2,3,5,6-tetraphenyl-1,4-dioxin (1)

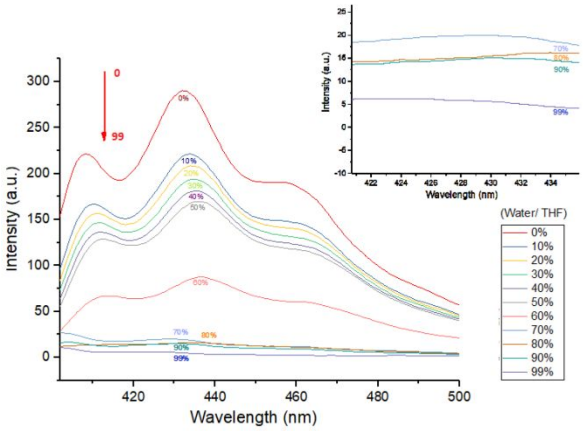

Figure S16. The fluorescence spectrum of 2,3,5,6-tetraphenyl dioxin (dioxin **1)** in THF/H_2O mixture.

2.5.2 Fluorescence spectrum of 2,3,4,5-tetraphenyl-4H-pyran-4-one (4)

After the synthesis of 2,3,4,5-tetraphenyl-4H-pyran-4-one (**4**), its fluorescence spectrum was collected to determine whether it shows ACQ or AIE property. Absorption maximum of 2,3,4,5-tetraphenyl-4H-pyran-4-one (**4**) is 260 nm in THF based on UV spectrum. Emission maximum is 307 nm in THF, although it is shifted as water percentage increases. Moreover, the concentration of all solutions is 10 µM, **Table S1** indicates how the water/THF solutions were prepared.

Table S1. The mixtures of 2,3,4,5-tetraphenyl-4H-pyran-4-one (4) in water/THF system

f	Stock Concentration (mol/L)	THF/mL	Water/mL	Standard Concentration (mol/L)
**0 %**	0.5 mL $1.0x10^{-4}$ mol/L in THF	4.5	0.0	5 mL, $1.0x10^{-5}$ mol/L in water/ THF
**10 %**		4.0	0.5	
**20 %**		3.5	1.0	
**30 %**		3.0	1.5	
**40 %**		2.5	2.0	
**50 %**		2.0	2.5	
**60 %**		1.5	3.0	
**70 %**		1.0	3.5	
**80 %**		0.5	4.0	
**90 %**		0.0	4.5	

Fluorescence spectra were collected with Agilent Technologies Cary Eclipse Fluorescence Spectrophotometer. The measurement parameters were set to be 5 nm excitation and emission slit with at 700 V for all samples. The samples were excited with 260 nm light, the emission spectrum maximum is at 307 nm. Also, emission spectra were collected in 280-800 nm portion of the spectrum, but the data between 280-400 nm were presented. **Figure S17** indicates the fluorescence spectrum with varying water/THF fractions. Here, the water fraction is presented as f% is on the graph, where f is the amount of water in the solutions with respect to THF.

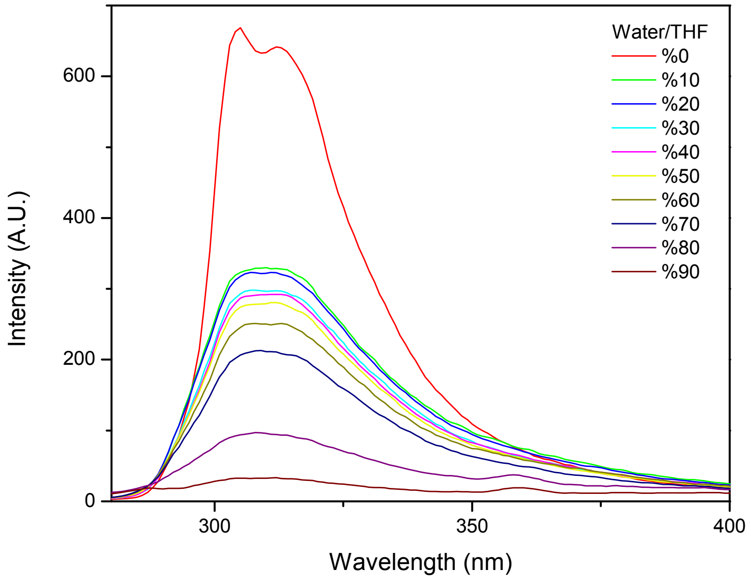

Figure S17. Fluorescence spectrum of 2,3,4,5-tetraphenyl-4H-pyran-4-one (**4**) with varying water %.

2.6 Quantum Yield Calculation for Dioxin 1

In this measurement, tyrosine was employed as standard and dioxin **1** is the sample. Fluorescence measurement was performed via Agilent Technologies Cary Eclipse Fluorescence Spectrophotometer and UV measurements were conducted by Jasco V-730 Spectrophotometer. The fluorometer settings was established as below for both the sample and the standard:
Excitation slit: 5 nm
Emission slit: 5 nm
Voltage: 625 Volt
Excitation wavelength: 275 nm
After data collection, the area under the curve was obtained via Integrate function in Origin software. Moreover, Fit linear with X error fitting function in Origin software was employed in integrated area versus absorbance graph which was illustrated in **Figure S18**.

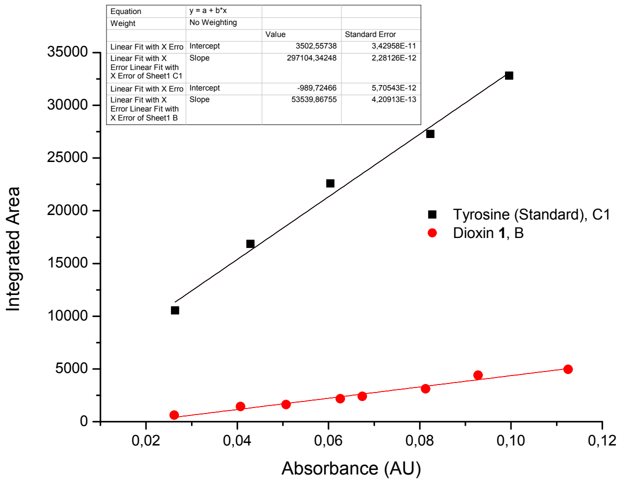

Figure S18. Integrated fluorescence spectrum area versus absorbance graph for dioxin **1**.

Calculation of quantum yield

The formula used as follows;

$$Qsample,\text{dioxine } \mathbf{1} = Qstd\left(\frac{msample}{mstd}\right)\left(\frac{\eta sample}{\eta std}\right)^2$$

Refractive index, η_{THF}=1.4073
m_{sample}=53539.86755
Refractive index, η_{water}=1.333
$m_{std,\ tyrosine}$=297104.34248
Qstd, tyrosine=0.13[4]

$$Qsample,\text{dioxine } \mathbf{1} = (0.13)\left(\frac{53539.86755}{297104.34248}\right)\left(\frac{1.4073}{1.333}\right)^2 = 0.026$$

2.7 Quantum Yield Calculation for 2,3,4,5-tetraphenyl-4H-pyran-4-one (4)

In this measurement, tyrosine was employed as standard and 2,3,4,5-tetraphenyl-4H-pyran-4-one (**4**) is the sample. Fluorescence measurement was performed via Agilent Technologies Cary Eclipse Fluorescence Spectrophotometer and UV measurements were conducted by Jasco V-730 Spectrophotometer. Both the sample and the standard at excited with 260 nm wavelength light at room temperature. The fluorometer settings was as below for both the sample and the standard:
Excitation slit: 2.5 nm
Emission slit: 5 nm
Voltage: 700 Volt
Excitation wavelength: 260 nm
After data collection, the area under the curve was obtained via Integrate function in Origin software. Moreover, Fit linear with X error fitting function in Origin software was employed in integrated area versus absorbance graph which was illustrated in **Figure S19**.

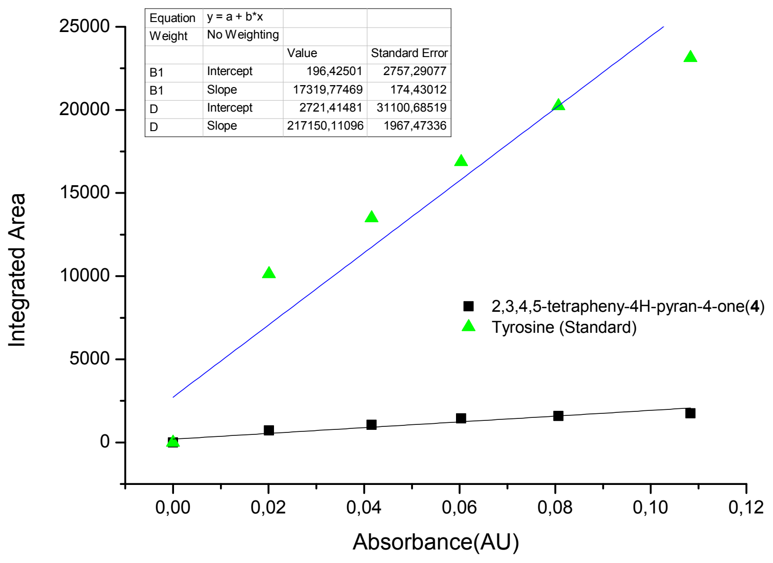

Figure S19. Integrated fluorescence spectrum area versus absorbance graph for 2,3,4,5-tetraphenyl-4H-pyran-4-one (**4**).

Calculation of quantum yield
The formula used as follows;

$$Qsample,\text{pyranone 4} = Qstd\left(\frac{msample,4}{mstd}\right)\left(\frac{\eta sample,4}{\eta std}\right)^2$$

Refractive index, η_{THF}=1.4073
m_{sample}=17319.77
Refractive index, η_{water}=1.333
$m_{std,\ tyrosine}$=217150.11
Qstd, tyrosine=0.13[4]

$$Qsample,\text{pyranone 4} = (0.13)\left(\frac{17319.77469}{217150.1109}\right)\left(\frac{1.4073}{1.333}\right)^2 = 0.011$$

2.8 Solid Fluorescence Analysis

Fluorescence measurement was carried out by Agilent Technologies Cary Eclipse Fluorescence Spectrophotometer. Approximately 18 mg of both dioxin **1** and pyranone **4** were weighed and placed into sample holder on the solid fluorescence apparatus, then the measurement was performed.

2.8.1 Solid fluorescence spectrum of dioxin 1

The parameters were set in the measurement as below.
Excitation slit: 5 nm
Emission slit: 5 nm

Voltage: 600 Volt
Excitation wavelength: 275 nm

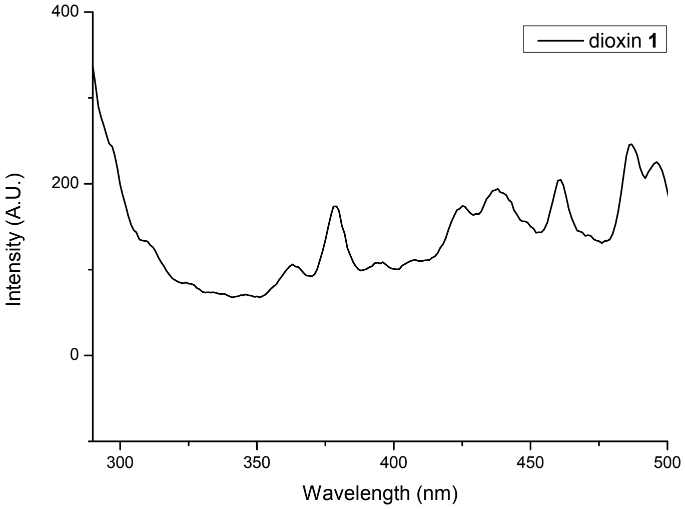

Figure S20. Solid fluorescence spectrum of dioxin **1** with 275 nm excitation wavelength.

2.8.2 Solid fluorescence of pyranone 4

The parameters were adjusted in the measurement as below.
Excitation slit: 5 nm
Emission slit: 5 nm
Voltage: 600 Volt
Excitation wavelength: 260 nm

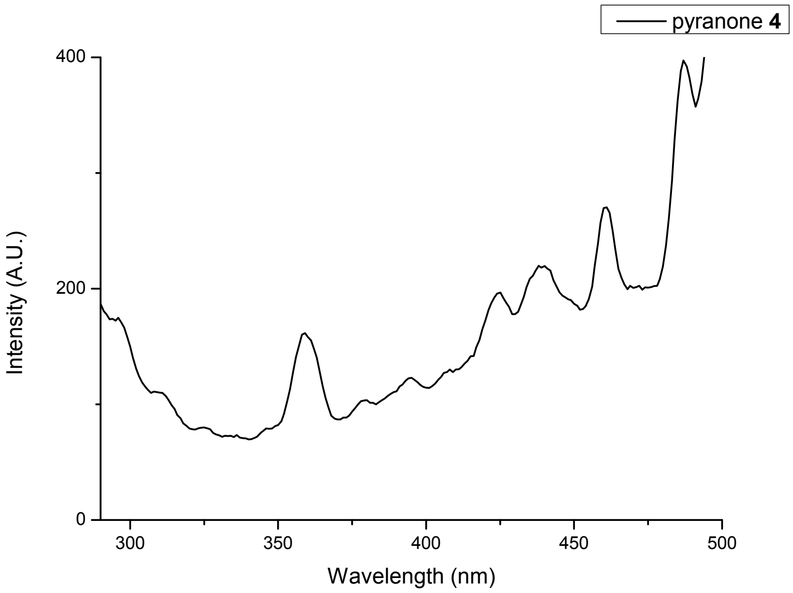

Figure S21. Solid fluorescence spectrum of pyranone **4** with 260 nm excitation wavelength.

2.9 XRD ANALYSIS

Selected data of single crystal X-ray diffraction (XRD) analysis was given in the following section.

2.9.1 XRD data of 2,3,5,6-tetraphenyl-1,4-dioxin (Dioxin 1)

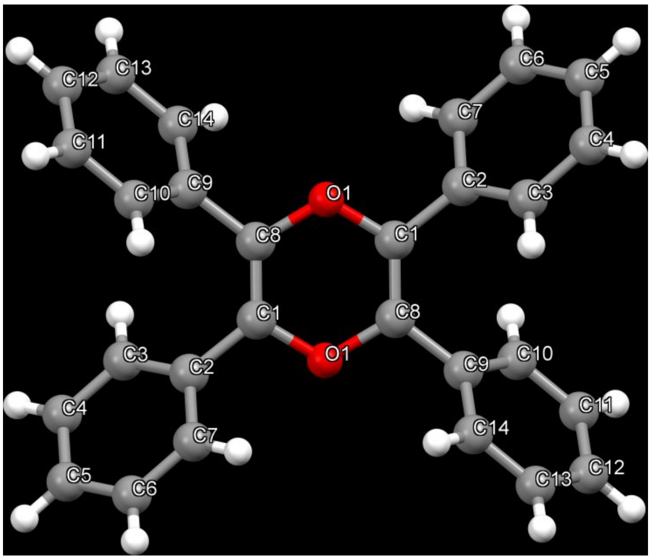

Figure S22. The crystal structure of dioxin **1**, showing the atom numbering scheme.

Table S2. Crystal data and structure refinement for dioxin **1.**

Identification code	Dioxin 1
Empirical formula	$C_{28}H_{20}O_2$
Formula weight	388.44
Temperature/K	153
Crystal system	monoclinic
Space group	$P2_1/n$
a/Å	12.743(4)
b/Å	5.4517(18)
c/Å	15.003(5)
α/°	90
β/°	103.257(7)
γ/°	90
Volume/Å^3	1014.5(6)
Z	2
ρ_{calc}g/cm^3	1.272
μ/mm^{-1}	0.079
F(000)	408.0
Crystal size/mm^3	0.2 × 0.18 × 0.04
Radiation	MoKα (λ = 0.71075)
2Θ range for data collection/°	6.57 to 49.998
Index ranges	-15 ≤ h ≤ 15, -6 ≤ k ≤ 6, -17 ≤ l ≤ 17
Reflections collected	18474
Independent reflections	1786 [R_{int} = 0.0533, R_{sigma} = 0.0315]
Data/restraints/parameters	1786/2/146
Goodness-of-fit on F^2	1.079
Final R indexes [I>=2σ (I)]	R_1 = 0.0349, wR_2 = 0.0851
Final R indexes [all data]	R_1 = 0.0385, wR_2 = 0.0871
Largest diff. peak/hole / e Å^{-3}	0.16/-0.21

Table S3. Fractional Atomic Coordinates (×10^4) and Equivalent Isotropic Displacement Parameters (Å^2×10^3) for dioxin **1**. U_{eq} is defined as 1/3 of of the trace of the orthogonalised U_{IJ} tensor.

**Atom**	***x***	***y***	***z***	**U(eq)**
O1	8935(9)	160(60)	4507(6)	38(3)
O2	8984(8)	1070(50)	4561(6)	47(2)
C1	9197.5(9)	37(3)	5459.8(8)	34.6(3)
C2	8209.4(9)	79(2)	5810.8(7)	25.5(3)
C3	7974.4(9)	-1775(2)	6375.0(8)	27.7(3)
C4	7015.6(9)	-1743(2)	6661.2(8)	30.1(3)
C5	6278.0(9)	130(2)	6385.0(8)	30.2(3)
C6	6501.1(9)	1971(2)	5821.6(8)	31.0(3)
C7	7456.9(9)	1946(2)	5532.7(8)	29.1(3)
C8	9813.2(9)	606(3)	4100.1(8)	34.9(3)
C9	9426.6(8)	1142(2)	3117.2(7)	25.9(3)
C10	9658.2(9)	-454(2)	2464.0(8)	27.3(3)
C11	9253.7(9)	-15(2)	1541.0(8)	30.7(3)
C12	8611.5(9)	2015(2)	1257.8(8)	32.0(3)
C13	8367.8(9)	3604(2)	1900.5(8)	33.5(3)
C14	8768.5(9)	3174(2)	2825.9(8)	31.5(3)

Table S4. Anisotropic Displacement Parameters (Å^2×10^3) for dioxin **1**. The Anisotropic displacement factor exponent takes the form: $-2\pi^2[h^2a^{*2}U_{11}+2hka^*b^*U_{12}+\ldots]$.

**Atom**	U_{11}	U_{22}	U_{33}	U_{23}	U_{13}	U_{12}
O1	18.5(17)	77(7)	18.2(18)	6(3)	5.6(12)	5(3)
O2	22.6(13)	99(7)	21.4(12)	14(2)	7.1(10)	15(3)
C1	24.5(6)	61.4(8)	18.0(6)	2.3(5)	5.0(5)	1.4(6)
C2	20.4(5)	36.3(6)	19.1(5)	-4.7(4)	2.9(4)	-2.1(5)
C3	23.8(6)	31.1(6)	26.7(6)	-1.4(5)	2.5(4)	2.4(5)
C4	27.0(6)	32.3(6)	31.5(6)	3.1(5)	7.9(5)	-3.6(5)
C5	21.9(6)	40.2(7)	30.1(6)	-1.1(5)	9.3(5)	-0.4(5)
C6	28.7(6)	35.3(7)	29.4(6)	1.6(5)	7.5(5)	7.4(5)
C7	30.3(6)	33.0(6)	24.7(6)	2.7(5)	7.9(5)	-0.9(5)
C8	21.0(6)	61.9(9)	23.2(6)	1.6(6)	8.2(5)	0.8(5)
C9	17.1(5)	37.7(6)	22.7(6)	2.3(5)	4.4(4)	-3.5(5)
C10	22.9(6)	31.9(6)	26.3(6)	2.6(5)	4.2(4)	-0.4(5)
C11	30.4(6)	37.7(7)	23.2(6)	-3.6(5)	4.3(5)	-4.8(5)
C12	27.2(6)	42.7(7)	23.1(6)	6.3(5)	-0.7(5)	-6.4(5)
C13	24.5(6)	35.5(7)	38.7(7)	9.0(5)	3.5(5)	1.7(5)
C14	25.0(6)	38.5(7)	31.8(6)	-2.8(5)	8.4(5)	0.3(5)

Table S5. Bond Lengths for dioxin **1**.

**Atom**	**Atom**	**Length/Å**	**Atom**	**Atom**	**Length/Å**
O1	C1	1.393(9)	C5	C6	1.3829(17)
O1	C8	1.413(9)	C6	C7	1.3834(16)
O2	C1	1.428(7)	C8	C9	1.4730(16)
O2	C8	1.412(6)	C9	C10	1.3919(16)
C1	C2	1.4722(16)	C9	C14	1.3977(17)
C1	C8^1	1.3289(17)	C10	C11	1.3832(16)
C2	C3	1.3944(16)	C11	C12	1.3838(17)
C2	C7	1.3948(16)	C12	C13	1.3836(18)
C3	C4	1.3850(16)	C13	C14	1.3850(17)
C4	C5	1.3854(17)			

[1]2-X,-Y,1-Z

Table S6. Bond Angles for dioxin **1**.

Atom	Atom	Atom	Angle/˚	Atom	Atom	Atom	Angle/˚
C1	O1	C8	115.1(7)	C6	C7	C2	120.52(11)
C8	O2	C1	113.0(6)	O1	C8	C9	110.5(5)
O1	C1	C2	110.0(5)	O2	C8	C9	109.8(2)
O2	C1	C2	109.8(2)	C1[1]	C8	O1	119.7(7)
C8[1]	C1	O1	120.0(7)	C1[1]	C8	O2	122.3(3)
C8[1]	C1	O2	121.8(3)	C1[1]	C8	C9	127.67(10)
C8[1]	C1	C2	128.17(11)	C10	C9	C8	120.30(11)
C3	C2	C1	121.75(10)	C10	C9	C14	118.97(10)
C3	C2	C7	118.80(10)	C14	C9	C8	120.62(11)
C7	C2	C1	119.35(10)	C11	C10	C9	120.40(11)
C4	C3	C2	120.44(10)	C10	C11	C12	120.29(11)
C3	C4	C5	120.19(11)	C13	C12	C11	119.88(11)
C6	C5	C4	119.82(11)	C12	C13	C14	120.16(11)
C5	C6	C7	120.23(11)	C13	C14	C9	120.30(11)

[1]2-X,-Y,1-Z

Table S7. Torsion Angles for dioxin **1**.

A	B	C	D	Angle/˚	A	B	C	D	Angle/˚
O1	C1	C2	C3	123.7(14)	C3	C4	C5	C6	0.02(17)
O1	C1	C2	C7	-52.6(14)	C4	C5	C6	C7	0.07(17)
O1	C8	C9	C10	-114.6(14)	C5	C6	C7	C2	-0.50(17)
O1	C8	C9	C14	61.4(14)	C7	C2	C3	C4	-0.73(16)
O2	C1	C2	C3	145.5(12)	C8	O1	C1	C2	168.4(17)
O2	C1	C2	C7	-30.9(12)	C8	O1	C1	C8[1]	-26(3)
O2	C8	C9	C10	-136.4(12)	C8	O2	C1	C2	-166.8(13)
O2	C8	C9	C14	39.7(12)	C8	O2	C1	C8[1]	19(2)
C1	O1	C8	C1[1]	26(3)	C8[1]	C1	C2	C3	-40.7(2)
C1	O1	C8	C9	-169.8(17)	C8[1]	C1	C2	C7	142.94(14)
C1	O2	C8	C1[1]	-19(2)	C8	C9	C10	C11	176.96(10)
C1	O2	C8	C9	165.5(13)	C8	C9	C14	C13	-177.04(11)
C1	C2	C3	C4	-177.10(11)	C9	C10	C11	C12	-0.19(17)
C1	C2	C7	C6	177.28(11)	C10	C9	C14	C13	-0.92(16)
C1[1]	C8	C9	C10	48.43(19)	C10	C11	C12	C13	-0.36(17)
C1[1]	C8	C9	C14	-135.50(15)	C11	C12	C13	C14	0.26(17)
C2	C3	C4	C5	0.32(17)	C12	C13	C14	C9	0.38(17)
C3	C2	C7	C6	0.83(16)	C14	C9	C10	C11	0.82(16)

[1]2-X,-Y,1-Z

Table S8. Hydrogen Atom Coordinates (Å×10^4) and Isotropic Displacement Parameters (Å^2×10^3) for dioxin **1**.

Atom	*x*	*y*	*z*	U(eq)
H3	8475.17	-3069.14	6564.22	33
H4	6863.25	-3008.57	7048.1	36
H5	5620.83	150.47	6582.13	36
H6	5996.31	3257.59	5632.41	37
H7	7601.92	3209.43	5141.75	35
H10	10096.74	-1853.88	2653.19	33
H11	9417.41	-1110.64	1099.64	37

Atom	*x*	*y*	*z*	U(eq)
H12	8338.44	2316.28	623.2	38
H13	7924.46	4994.25	1706.5	40
H14	8595.34	4265.39	3264.62	38

Table S9. Atomic Occupancy for dioxin **1**.

Atom	*Occupancy*	Atom	*Occupancy*	Atom	*Occupancy*
O1	0.36(6)	O2	0.64(6)		

Experimental

Single crystals of $C_{28}H_{20}O_2$ [Dioxin **1**] were obtained in the solution of toluene and petroleum ether. Dioxin **1** was first dissolved in petroleum ether by gentle heating up to complete dissolution, then toluene was dropped until forming a turbid solution. Moreover, the turbid solution was boiled via a heating mantle. Finally, the solution was cooled at room temperature and was kept in a closed container for 4 days. A suitable yellow crystal was selected and analyzed on a Rigaku RAXIS conversion diffractometer. The crystal was kept at 153 K during data collection. Using Olex2 [1], the structure was solved with the SHELXT [2] structure solution program using Intrinsic Phasing and refined with the SHELXL [3] refinement package using Least Squares minimization.

1. Dolomanov, O.V., Bourhis, L.J., Gildea, R.J, Howard, J.A.K. & Puschmann, H. (2009), J. Appl. Cryst. 42, 339-341.

Crystal structure determination of dioxin 1

Crystal Data for $C_{28}H_{20}O_2$ (M =388.44 g/mol): monoclinic, space group P2$_1$/n (no. 14), a = 12.743(4) Å, b = 5.4517(18) Å, c = 15.003(5) Å, β = 103.257(7)°, V = 1014.5(6) Å^3, Z = 2, T = 153 K, μ(MoKα) = 0.079 mm^{-1}, $Dcalc$ = 1.272 g/cm^3, 18474 reflections measured (6.57° ≤ 2Θ ≤ 49.998°), 1786 unique (R_{int} = 0.0533, R_{sigma} = 0.0315) which were used in all calculations. The final R_1 was 0.0349 (I > 2σ(I)) and wR_2 was 0.0871 (all data).

Refinement model description

Number of restraints - 2, number of constraints - unknown.
Details:
1. Restrained distances
O1-C8 ≈ O2-C8
with sigma of 0.02
O1-C1 ≈ O2-C1
with sigma of 0.02
2. Others
Sof(O2)=1-FVAR(1)
Sof(O1)=FVAR(1)
3.a Aromatic/amide H refined with riding coordinates:
C3(H3), C4(H4), C5(H5), C6(H6), C7(H7), C10(H10), C11(H11), C12(H12),
C13(H13), C14(H14)

This report has been created with Olex2, compiled on 2020.11.12 svn.r5f609507 for OlexSys. Please let us know if there are any errors or if you would like to have additional features.

2.9.2 XRD data of 2,3,4,5-tetraphenyl-4H-pyran-4-one (4)

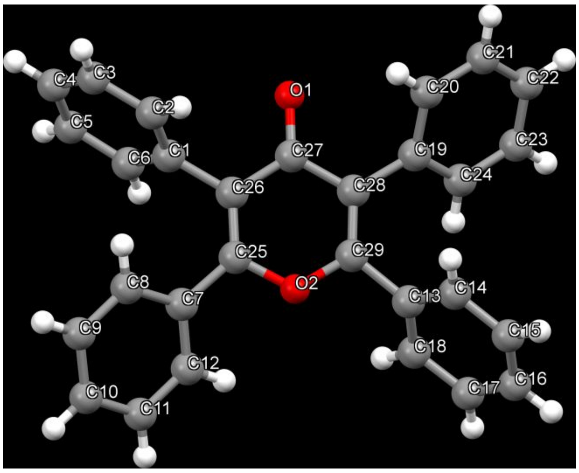

Figure S23. The crystal structure of pyranone **4**, showing the atom numbering scheme.

Table S10. Crystal data and structure refinement for 2,3,4,5-tetraphenyl-4H-pyran-4-one (**4**).

Identification code	2,3,4,5-tetraphenyl-4H-pyran-4-one (pyranone **4**)
Empirical formula	$C_{29}H_{20}O_2$
Formula weight	400.45
Temperature/K	298
Crystal system	monoclinic
Space group	$P2_1/n$
a/Å	11.866(8)
b/Å	11.674(8)
c/Å	14.828(10)
α/°	90
β/°	93.053(14)
γ/°	90
Volume/Å^3	2051(2)
Z	4
ρ_{calc}g/cm^3	1.297
μ/mm^{-1}	0.080
F(000)	840.0
Crystal size/mm^3	0.353 × 0.188 × 0.106
Radiation	MoKα (λ = 0.71073)
2Θ range for data collection/°	4.286 to 50.054
Index ranges	-14 ≤ h ≤ 14, -13 ≤ k ≤ 13, -17 ≤ l ≤ 17
Reflections collected	23804
Independent reflections	3606 [R_{int} = 0.0879, R_{sigma} = 0.0655]
Data/restraints/parameters	3606/0/281
Goodness-of-fit on F^2	1.061
Final R indexes [I>=2σ (I)]	R_1 = 0.0830, wR_2 = 0.2179
Final R indexes [all data]	R_1 = 0.1337, wR_2 = 0.2430
Largest diff. peak/hole / e Å^{-3}	0.51/-0.27

Table S11. Fractional Atomic Coordinates (×10^4) and Equivalent Isotropic Displacement Parameters (Å^2×10^3) for pyranone **4**. U_{eq} is defined as 1/3 of of the trace of the orthogonalised U_{IJ} tensor.

Atom	*x*	*y*	*z*	U(eq)
O(1)	6052(3)	4165(3)	3517(3)	72.0(12)
O(2)	8043(3)	5411(2)	5575(2)	43.4(8)
C(1)	7612(4)	2637(4)	4318(3)	36.1(10)
C(2)	6795(4)	1844(4)	4505(3)	44.4(12)
C(3)	6891(5)	726(4)	4191(3)	53.9(13)
C(4)	7773(5)	426(4)	3684(3)	56.9(14)
C(5)	8582(4)	1221(4)	3482(4)	53.4(13)
C(6)	8504(4)	2330(4)	3816(3)	48.8(12)
C(7)	9077(4)	3733(4)	5879(3)	39.8(11)
C(8)	9020(4)	2603(4)	6159(3)	46.7(12)
C(9)	9870(5)	2133(5)	6707(3)	59.2(15)
C(10)	10807(4)	2800(5)	6978(3)	53.5(14)
C(11)	10867(4)	3918(5)	6705(3)	54.1(13)
C(12)	10021(4)	4380(4)	6168(3)	48.4(12)
C(13)	7297(4)	7258(4)	5574(3)	37.4(11)
C(14)	6387(4)	7668(4)	6016(3)	50.7(13)
C(15)	6434(5)	8765(4)	6387(4)	59.3(15)
C(16)	7375(5)	9427(4)	6292(4)	59.4(15)
C(17)	8287(5)	9009(4)	5867(4)	57.7(14)
C(18)	8258(4)	7915(4)	5505(3)	43.9(12)
C(19)	5732(4)	6485(4)	3969(3)	38.8(11)
C(20)	4633(4)	6108(5)	3722(3)	50.6(13)
C(21)	3902(4)	6813(6)	3207(3)	59.3(15)
C(22)	4213(5)	7867(6)	2942(4)	67.7(17)
C(23)	5289(5)	8255(5)	3180(3)	61.7(15)
C(24)	6036(4)	7570(4)	3689(3)	48.3(12)
C(25)	8165(4)	4281(3)	5321(3)	36.5(10)
C(26)	7504(4)	3844(4)	4648(3)	35.9(10)
C(27)	6655(4)	4548(4)	4181(3)	40.4(11)
C(28)	6551(4)	5730(4)	4470(3)	36.4(10)
C(29)	7247(4)	6094(3)	5156(3)	36.6(10)

Table S12. Anisotropic Displacement Parameters (Å^2×10^3) for pyranone **4**. The Anisotropic displacement factor exponent takes the form: $-2\pi^2[h^2a^{*2}U_{11}+2hka^*b^*U_{12}+\ldots]$.

Atom	U_{11}	U_{22}	U_{33}	U_{23}	U_{13}	U_{12}
O(1)	86(3)	56(2)	69(3)	-16(2)	-39(2)	14(2)
O(2)	53(2)	31.3(17)	44.4(18)	-6.3(14)	-10.4(15)	1.4(14)
C(1)	41(3)	32(2)	34(2)	-1.2(19)	-9(2)	2(2)
C(2)	50(3)	45(3)	38(3)	-7(2)	-2(2)	-3(2)
C(3)	71(4)	39(3)	51(3)	0(2)	-2(3)	-11(3)
C(4)	82(4)	34(3)	53(3)	-6(2)	-14(3)	11(3)
C(5)	59(3)	45(3)	56(3)	-14(2)	1(3)	9(3)
C(6)	53(3)	43(3)	50(3)	-3(2)	0(2)	0(2)
C(7)	44(3)	39(3)	36(2)	-3(2)	-2(2)	-2(2)
C(8)	51(3)	42(3)	46(3)	0(2)	-10(2)	4(2)
C(9)	70(4)	52(3)	53(3)	1(3)	-13(3)	16(3)
C(10)	50(3)	69(4)	41(3)	0(3)	-11(2)	17(3)
C(11)	48(3)	67(4)	47(3)	-1(3)	-5(2)	-7(3)
C(12)	54(3)	50(3)	40(3)	0(2)	-5(2)	-7(2)
C(13)	46(3)	30(2)	35(2)	-1.4(19)	-8(2)	5(2)
C(14)	51(3)	48(3)	52(3)	-9(2)	-2(2)	7(2)
C(15)	69(4)	46(3)	64(3)	-17(3)	8(3)	13(3)
C(16)	87(4)	31(3)	60(3)	-10(2)	-11(3)	12(3)

Atom	U_{11}	U_{22}	U_{33}	U_{23}	U_{13}	U_{12}
C(17)	71(4)	40(3)	61(3)	-2(3)	-6(3)	-8(3)
C(18)	48(3)	39(3)	44(3)	-7(2)	0(2)	-3(2)
C(19)	41(3)	41(3)	34(2)	-6(2)	-6(2)	0(2)
C(20)	49(3)	61(3)	42(3)	-1(2)	3(2)	-2(3)
C(21)	45(3)	86(4)	45(3)	-14(3)	-11(2)	12(3)
C(22)	74(4)	80(4)	47(3)	-12(3)	-13(3)	32(4)
C(23)	86(4)	48(3)	50(3)	-2(3)	-12(3)	16(3)
C(24)	54(3)	44(3)	45(3)	-6(2)	-11(2)	3(2)
C(25)	46(3)	25(2)	39(2)	-0.1(19)	0(2)	1.1(19)
C(26)	42(3)	29(2)	36(2)	-2.4(19)	0(2)	-2.1(19)
C(27)	50(3)	30(2)	40(3)	-6(2)	-1(2)	-1(2)
C(28)	46(3)	29(2)	34(2)	-5.2(19)	3(2)	-5(2)
C(29)	42(3)	26(2)	41(3)	2.2(19)	-1(2)	5(2)

Table S13. Bond Lengths for pyranone **4**.

Atom	Atom	Length/Å	Atom	Atom	Length/Å
O(1)	C(27)	1.268(5)	C(13)	C(18)	1.382(6)
O(2)	C(25)	1.381(5)	C(13)	C(29)	1.493(6)
O(2)	C(29)	1.361(5)	C(14)	C(15)	1.393(7)
C(1)	C(2)	1.379(6)	C(15)	C(16)	1.371(8)
C(1)	C(6)	1.374(6)	C(16)	C(17)	1.370(7)
C(1)	C(26)	1.499(6)	C(17)	C(18)	1.385(7)
C(2)	C(3)	1.392(7)	C(19)	C(20)	1.407(6)
C(3)	C(4)	1.366(7)	C(19)	C(24)	1.386(7)
C(4)	C(5)	1.380(7)	C(19)	C(28)	1.481(6)
C(5)	C(6)	1.391(7)	C(20)	C(21)	1.394(7)
C(7)	C(8)	1.385(6)	C(21)	C(22)	1.349(8)
C(7)	C(12)	1.399(6)	C(22)	C(23)	1.383(8)
C(7)	C(25)	1.473(6)	C(23)	C(24)	1.387(7)
C(8)	C(9)	1.375(7)	C(25)	C(26)	1.337(6)
C(9)	C(10)	1.399(7)	C(26)	C(27)	1.448(6)
C(10)	C(11)	1.370(7)	C(27)	C(28)	1.452(6)
C(11)	C(12)	1.360(7)	C(28)	C(29)	1.346(6)
C(13)	C(14)	1.379(6)			

Table S14. Bond Angles for pyranone **4**.

Atom	Atom	Atom	Angle/˚	Atom	Atom	Atom	Angle/˚
C(29)	O(2)	C(25)	121.0(3)	C(13)	C(18)	C(17)	119.1(5)
C(2)	C(1)	C(26)	119.5(4)	C(20)	C(19)	C(28)	121.2(4)
C(6)	C(1)	C(2)	120.3(4)	C(24)	C(19)	C(20)	117.4(4)
C(6)	C(1)	C(26)	120.2(4)	C(24)	C(19)	C(28)	121.3(4)
C(1)	C(2)	C(3)	119.4(5)	C(21)	C(20)	C(19)	119.9(5)
C(4)	C(3)	C(2)	120.2(5)	C(22)	C(21)	C(20)	121.8(5)
C(3)	C(4)	C(5)	120.6(5)	C(21)	C(22)	C(23)	119.2(5)
C(4)	C(5)	C(6)	119.3(5)	C(22)	C(23)	C(24)	120.3(5)
C(1)	C(6)	C(5)	120.2(5)	C(19)	C(24)	C(23)	121.4(5)
C(8)	C(7)	C(12)	118.2(4)	O(2)	C(25)	C(7)	110.2(4)
C(8)	C(7)	C(25)	122.4(4)	C(26)	C(25)	O(2)	120.2(4)
C(12)	C(7)	C(25)	119.3(4)	C(26)	C(25)	C(7)	129.6(4)
C(9)	C(8)	C(7)	120.8(5)	C(25)	C(26)	C(1)	123.1(4)

Atom	Atom	Atom	Angle/°	Atom	Atom	Atom	Angle/°
C(8)	C(9)	C(10)	119.6(5)	C(25)	C(26)	C(27)	120.4(4)
C(11)	C(10)	C(9)	119.9(5)	C(27)	C(26)	C(1)	116.5(4)
C(12)	C(11)	C(10)	120.2(5)	O(1)	C(27)	C(26)	121.4(4)
C(11)	C(12)	C(7)	121.3(5)	O(1)	C(27)	C(28)	120.7(4)
C(14)	C(13)	C(18)	120.8(4)	C(26)	C(27)	C(28)	117.8(4)
C(14)	C(13)	C(29)	119.9(4)	C(27)	C(28)	C(19)	118.8(4)
C(18)	C(13)	C(29)	119.3(4)	C(29)	C(28)	C(19)	123.5(4)
C(13)	C(14)	C(15)	119.3(5)	C(29)	C(28)	C(27)	117.7(4)
C(16)	C(15)	C(14)	119.6(5)	O(2)	C(29)	C(13)	109.4(4)
C(17)	C(16)	C(15)	120.9(5)	C(28)	C(29)	O(2)	122.9(4)
C(16)	C(17)	C(18)	120.1(5)	C(28)	C(29)	C(13)	127.6(4)

Table S15. Torsion Angles for pyranone **4.**

A	B	C	D	Angle/°	A	B	C	D	Angle/°
O(1)	C(27)	C(28)	C(19)	-0.5(7)	C(16)	C(17)	C(18)	C(13)	0.3(7)
O(1)	C(27)	C(28)	C(29)	-177.4(4)	C(18)	C(13)	C(14)	C(15)	0.7(7)
O(2)	C(25)	C(26)	C(1)	178.2(4)	C(18)	C(13)	C(29)	O(2)	64.2(5)
O(2)	C(25)	C(26)	C(27)	-0.1(6)	C(18)	C(13)	C(29)	C(28)	-115.4(5)
C(1)	C(2)	C(3)	C(4)	-1.5(7)	C(19)	C(20)	C(21)	C(22)	-0.9(8)
C(1)	C(26)	C(27)	O(1)	-1.3(7)	C(19)	C(28)	C(29)	O(2)	-175.8(4)
C(1)	C(26)	C(27)	C(28)	-178.4(4)	C(19)	C(28)	C(29)	C(13)	3.6(7)
C(2)	C(1)	C(6)	C(5)	0.8(7)	C(20)	C(19)	C(24)	C(23)	-0.4(7)
C(2)	C(1)	C(26)	C(25)	108.3(5)	C(20)	C(19)	C(28)	C(27)	44.8(6)
C(2)	C(1)	C(26)	C(27)	-73.4(5)	C(20)	C(19)	C(28)	C(29)	-138.4(5)
C(2)	C(3)	C(4)	C(5)	0.4(8)	C(20)	C(21)	C(22)	C(23)	0.7(8)
C(3)	C(4)	C(5)	C(6)	1.3(8)	C(21)	C(22)	C(23)	C(24)	-0.4(8)
C(4)	C(5)	C(6)	C(1)	-2.0(8)	C(22)	C(23)	C(24)	C(19)	0.3(8)
C(6)	C(1)	C(2)	C(3)	0.9(7)	C(24)	C(19)	C(20)	C(21)	0.7(7)
C(6)	C(1)	C(26)	C(25)	-73.2(6)	C(24)	C(19)	C(28)	C(27)	-132.1(5)
C(6)	C(1)	C(26)	C(27)	105.1(5)	C(24)	C(19)	C(28)	C(29)	44.6(6)
C(7)	C(8)	C(9)	C(10)	-0.7(8)	C(25)	O(2)	C(29)	C(13)	179.3(4)
C(7)	C(25)	C(26)	C(1)	-1.4(7)	C(25)	O(2)	C(29)	C(28)	-1.1(6)
C(7)	C(25)	C(26)	C(27)	-179.6(4)	C(25)	C(7)	C(8)	C(9)	-177.6(4)
C(8)	C(7)	C(12)	C(11)	0.3(7)	C(25)	C(7)	C(12)	C(11)	178.1(4)
C(8)	C(7)	C(25)	O(2)	143.2(4)	C(25)	C(26)	C(27)	O(1)	177.0(5)
C(8)	C(7)	C(25)	C(26)	-37.2(7)	C(25)	C(26)	C(27)	C(28)	-0.1(6)
C(8)	C(9)	C(10)	C(11)	0.8(8)	C(26)	C(1)	C(2)	C(3)	179.4(4)
C(9)	C(10)	C(11)	C(12)	-0.3(8)	C(26)	C(1)	C(6)	C(5)	-177.7(4)
C(10)	C(11)	C(12)	C(7)	-0.2(8)	C(26)	C(27)	C(28)	C(19)	176.6(4)
C(12)	C(7)	C(8)	C(9)	0.2(7)	C(26)	C(27)	C(28)	C(29)	-0.3(6)
C(12)	C(7)	C(25)	O(2)	-34.5(6)	C(27)	C(28)	C(29)	O(2)	0.9(6)
C(12)	C(7)	C(25)	C(26)	145.0(5)	C(27)	C(28)	C(29)	C(13)	-179.6(4)
C(13)	C(14)	C(15)	C(16)	1.3(8)	C(28)	C(19)	C(20)	C(21)	-176.4(4)
C(14)	C(13)	C(18)	C(17)	-1.5(7)	C(28)	C(19)	C(24)	C(23)	176.6(4)
C(14)	C(13)	C(29)	O(2)	-116.4(5)	C(29)	O(2)	C(25)	C(7)	-179.7(4)
C(14)	C(13)	C(29)	C(28)	64.0(6)	C(29)	O(2)	C(25)	C(26)	0.6(6)
C(14)	C(15)	C(16)	C(17)	-2.5(8)	C(29)	C(13)	C(14)	C(15)	-178.7(4)
C(15)	C(16)	C(17)	C(18)	1.7(8)	C(29)	C(13)	C(18)	C(17)	177.9(4)

Table S16. Hydrogen Atom Coordinates (Å×10^4) and Isotropic Displacement Parameters (Å^2×10^3) for pyranone **4.**

Atom	*x*	*y*	*z*	U(eq)
H(2)	6185	2054	4838	53
H(3)	6353	182	4326	65
H(4)	7828	-322	3474	68
H(5)	9173	1017	3127	64
H(6)	9058	2865	3699	59
H(8)	8401	2158	5973	56
H(9)	9821	1376	6897	71
H(10)	11389	2484	7343	64
H(11)	11488	4362	6888	65
H(12)	10070	5141	5989	58
H(14)	5747	7217	6066	61
H(15)	5832	9046	6697	71
H(16)	7395	10170	6519	71
H(17)	8927	9461	5822	69
H(18)	8876	7627	5219	53
H(20)	4394	5388	3902	61
H(21)	3181	6550	3041	71
H(22)	3710	8328	2604	81
H(23)	5513	8979	2997	74
H(24)	6756	7843	3846	58

Experimental

Single crystals of $C_{29}H_{20}O_2$ [2,3,4,5-tetraphenyl-4H-pyran-4-one(**4**)] were gathered in the solution of ethanol: DCM(0.9:0.1) solvent system. Pyranone **4** was recrystallized repeatedly with an ethanol-DCM solvent system until obtaining a highly pure product. Then, the product was recrystallized once more and cooled at room temperature. The crystallization was completed at room temperature by keeping the product in a closed container until all the solvent was evaporated. A suitable white crystal was selected and analyzed on a Bruker APEX II QUAZAR three-circle diffractometer. The crystal was kept at 298 K during data collection. Using Olex2 [1], the structure was solved with the XT [2] structure solution program using Intrinsic Phasing and refined with the XL [3] refinement package using Least Squares minimization.

1. Dolomanov, O.V., Bourhis, L.J., Gildea, R.J, Howard, J.A.K. & Puschmann, H. (2009), J. Appl. Cryst. 42, 339-341.
2. Sheldrick, G.M. (2015). Acta Cryst. A71, 3-8.
3. Sheldrick, G.M. (2008). Acta Cryst. A64, 112-122.

Crystal structure determination of pyranone 4

Crystal Data for $C_{29}H_{20}O_2$ (M =400.45 g/mol): monoclinic, space group $P2_1/n$ (no. 14), a = 11.866(8) Å, b = 11.674(8) Å, c = 14.828(10) Å, β = 93.053(14)°, V = 2051(2) Å^3, Z = 4, T = 298 K, μ(MoKα) = 0.080 mm^{-1}, $Dcalc$ = 1.297 g/cm^3, 23804 reflections measured (4.286° ≤ 2Θ ≤ 50.054°), 3606 unique (R_{int} = 0.0879, R_{sigma} = 0.0655) which were used in all calculations. The final R_1 was 0.0830 (I > 2σ(I)) and wR_2 was 0.2430 (all data).

Refinement model description

Number of restraints - 0, number of constraints - unknown.
Details:
1. Fixed Uiso
At 1.2 times of:
All C(H) groups
2.a Aromatic/amide H refined with riding coordinates:
C2(H2), C3(H3), C4(H4), C5(H5), C6(H6), C8(H8), C9(H9), C10(H10), C11(H11), C12(H12), C14(H14), C15(H15), C16(H16), C17(H17), C18(H18), C20(H20), C21(H21), C22(H22), C23(H23), C24(H24)

2.10 Theoretical studies

All calculations were performed with Orca 5.0.3.[5] Geometry optimizations for the benchmark were performed in the gas phase with the same functional and basis set as used for the corresponding TDDFT calculation, which involved implicit THF as a solvent. All other geometry optimizations and TDDFT calculations presented in this work were performed at the (TD-)ωB97X-D3/def2-SVP/CPCM(THF) level. Vibrational frequencies were systematically calculated (analytically for ground states and numerically for excited states) to characterize the structures as true minima. Two spectra of TPD were calculated for an ensemble of 400 structures each obtained as Wigner distributions at 300K, by sampling the normal modes of butterfly and propeller conformations of S0 and S2 states, to simulate absorption and emission, respectively. The Wigner distributions of geometries were obtained using the tools distributed with the SHARC software.[6] Representations of molecular geometries and orbitals were done with the iboView software[7] using a threshold of 75 when drawing isosurfaces.

Commented [AM1]: Neese, Frank; Wennmohs, Frank; Becker, Ute; Riplinger, Christoph *Journal of Chemical Physics (2020), 152 (22), 224108CODEN: JCPSA6; ISSN:0021-9606. (American Institute of Physics)*

Commented [AM2]: 3 references to cite here:
Mai, S.; Richter, M.; Heindl, M.; Menger, M. F. S. J.; Atkins, A.; Ruckenbauer, M.; Plasser, F.; Ibele, L.M.; Kropf, S.; Oppel, M.; P. Marquetand, L. G.*SHARC2.1: Surface Hopping Including Arbitrary Couplings – Program Package for Non-Adiabatic Dynamics*. sharc-md.org

Richter, M.; Marquetand, P.; González-Vázquez, J.; Sola, I.; González, L. SHARC: Ab Initio Molecular Dynamics with Surface Hopping in the Adiabatic Representation Including Arbitrary Couplings. *J. Chem. Theory Comput.* **2011**, *7* (5), 1253– 1258, DOI: 10.1021/ct1007394

Mai, S.; Marquetand, P.; González, L. Nonadiabatic Dynamics: The SHARC Approach. *Wiley Interdiscip. Rev. Comput. Mol. Sci.* **2018**, *8* (6), e1370, DOI: 10.1002/wcms.1370

Commented [AM3]: 2 papers to cite here:
G. Knizia, Intrinsic atomic orbitals: An unbiased bridge between quantum theory and chemical concepts J. Chem. Theory Comput., 9 4834 (2013)

G. Knizia, J.E.M.N. Klein, Electron flow in reaction mechanisms --- revealed from first principles Angew. Chem. Int. Ed. 54 5518 (2015)

Table S17. Benchmark of density functionals and basis sets for the first six singlet states of TPD. The energy of the transition is given in electron volt and the oscillator strength is in parenthesis. According to the experimental absorption spectrum of TPP in THF, there should be no absorption above 300-350 nm (i.e., not bright state is expected bellow 3.5 eV). Only M06-2X, and the two range-separated functionals CAM-B3LYP, and wB97X-D3 seem valid for this system. The others tend to over-stabilize some dark and/or bright states, although there is, admittedly, no experimental support for the position of dark states.

functional	basis set	S1	S2	S3	S4	S5	S6
**bp86**	**SVP**	2.201(0.00)	2.613(0.06)	3.096(0.01)	3.116(0.00)	3.324(0.45)	3.456(0.00)
**bp86**	**TZVP**	2.213(0.00)	2.580(0.06)	3.036(0.01)	3.079(0.00)	3.270(0.45)	3.417(0.00)
**bp86**	**ma-SVP**	2.242(0.00)	2.617(0.06)	3.080(0.01)	3.130(0.00)	3.291(0.45)	3.460(0.00)
**bp86**	**ma-TZVP**	2.218(0.00)	2.578(0.07)	3.024(0.01)	3.076(0.00)	3.264(0.45)	3.414(0.00)
**pbe**	**SVP**	2.160(0.00)	2.591(0.06)	3.068(0.01)	3.088(0.00)	3.329(0.45)	3.419(0.00)
**pbe**	**TZVP**	2.173(0.00)	2.557(0.07)	3.006(0.01)	3.051(0.00)	3.275(0.44)	3.381(0.00)
**pbe**	**ma-SVP**	2.202(0.00)	2.591(0.07)	3.039(0.01)	3.096(0.00)	3.294(0.44)	3.419(0.00)
**pbe**	**ma-TZVP**	2.178(0.00)	2.552(0.07)	2.984(0.01)	3.043(0.00)	3.266(0.44)	3.375(0.00)
**tpss**	**SVP**	2.333(0.00)	2.750(0.06)	3.274(0.00)	3.295(0.00)	3.455(0.49)	3.632(0.00)
**tpss**	**TZVP**	2.344(0.00)	2.714(0.07)	3.207(0.01)	3.251(0.00)	3.401(0.49)	3.591(0.00)
**tpss**	**ma-SVP**	2.372(0.00)	2.748(0.07)	3.240(0.01)	3.297(0.00)	3.421(0.49)	3.624(0.02)
**tpss**	**ma-TZVP**	2.348(0.00)	2.709(0.07)	3.183(0.01)	3.242(0.00)	3.392(0.48)	3.580(0.02)
**m06L**	**SVP**	2.384(0.00)	2.870(0.08)	3.340(0.01)	3.352(0.00)	3.613(0.51)	3.711(0.00)
**m06L**	**TZVP**	2.366(0.00)	2.822(0.09)	3.263(0.01)	3.291(0.00)	3.570(0.50)	3.648(0.00)
**m06L**	**ma-SVP**	2.418(0.00)	2.871(0.09)	3.317(0.01)	3.355(0.00)	3.585(0.51)	3.711(0.00)
**m06L**	**ma-TZVP**	2.371(0.00)	2.820(0.09)	3.246(0.01)	3.285(0.00)	3.565(0.49)	3.643(0.00)
**b3lyp**	**SVP**	2.799(0.00)	3.357(0.17)	3.936(0.26)	3.975(0.24)	3.984(0.01)	4.286(0.01)
**b3lyp**	**TZVP**	2.803(0.00)	3.296(0.18)	3.840(0.02)	3.890(0.45)	3.906(0.00)	4.193(0.02)
**b3lyp**	**ma-SVP**	2.835(0.00)	3.335(0.19)	3.857(0.01)	3.908(0.46)	3.950(0.00)	4.219(0.02)
**b3lyp**	**ma-TZVP**	2.806(0.00)	3.284(0.19)	3.787(0.00)	3.876(0.46)	3.885(0.01)	4.164(0.01)
**pbe0**	**SVP**	2.913(0.00)	3.514(0.21)	4.105(0.37)	4.168(0.11)	4.184(0.00)	4.473(0.03)
**pbe0**	**TZVP**	2.912(0.00)	3.446(0.22)	4.019(0.09)	4.062(0.37)	4.092(0.00)	4.372(0.03)
**pbe0**	**ma-SVP**	2.948(0.00)	3.489(0.23)	4.044(0.08)	4.085(0.38)	4.144(0.00)	4.404(0.03)
**pbe0**	**ma-TZVP**	2.914(0.00)	3.433(0.23)	3.970(0.01)	4.045(0.44)	4.071(0.00)	4.348(0.02)
**tpssh**	**SVP**	2.632(0.00)	3.117(0.11)	3.700(0.01)	3.726(0.00)	3.759(0.52)	4.043(0.01)
**tpssh**	**TZVP**	2.639(0.00)	3.070(0.12)	3.610(0.00)	3.662(0.00)	3.697(0.51)	3.963(0.01)
**tpssh**	**ma-SVP**	2.669(0.00)	3.107(0.12)	3.641(0.00)	3.712(0.00)	3.719(0.51)	3.994(0.01)
**tpssh**	**ma-TZVP**	2.642(0.00)	3.062(0.12)	3.575(0.00)	3.648(0.00)	3.687(0.50)	3.947(0.01)
**m062x**	**SVP**	3.327(0.00)	4.088(0.43)	4.785(0.24)	4.857(0.04)	4.860(0.04)	5.139(0.06)
**m062x**	**TZVP**	3.346(0.00)	4.010(0.43)	4.664(0.04)	4.737(0.00)	4.769(0.26)	5.047(0.06)
**m062x**	**ma-SVP**	3.357(0.00)	4.024(0.42)	4.583(0.05)	4.753(0.01)	4.772(0.25)	5.034(0.01)
**m062x**	**ma-TZVP**	3.347(0.00)	3.980(0.41)	4.504(0.04)	4.692(0.00)	4.754(0.27)	4.860(0.00)
**cam-b3lyp**	**SVP**	3.351(0.00)	4.139(0.46)	4.848(0.14)	4.881(0.02)	4.892(0.15)	5.166(0.07)
**cam-b3lyp**	**TZVP**	3.358(0.00)	4.044(0.46)	4.696(0.03)	4.758(0.00)	4.813(0.26)	5.059(0.07)
**cam-b3lyp**	**ma-SVP**	3.397(0.00)	4.087(0.46)	4.662(0.04)	4.792(0.00)	4.840(0.25)	5.082(0.02)
**cam-b3lyp**	**ma-TZVP**	3.363(0.00)	4.019(0.44)	4.570(0.04)	4.722(0.00)	4.802(0.27)	5.013(0.01)
**ωb97x-d3**	**SVP**	3.540(0.00)	4.373(0.52)	5.081(0.00)	5.093(0.00)	5.220(0.23)	5.300(0.01)
**ωb97x-d3**	**TZVP**	3.547(0.00)	4.276(0.51)	4.945(0.00)	4.972(0.00)	5.143(0.20)	5.215(0.02)
**ωb97x-d3**	**ma-SVP**	3.591(0.00)	4.328(0.51)	4.949(0.02)	5.013(0.00)	5.161(0.19)	5.243(0.01)
**ωb97x-d3**	**ma-TZVP**	3.551(0.00)	4.255(0.49)	4.859(0.03)	4.947(0.00)	5.115(0.20)	5.202(0.02)

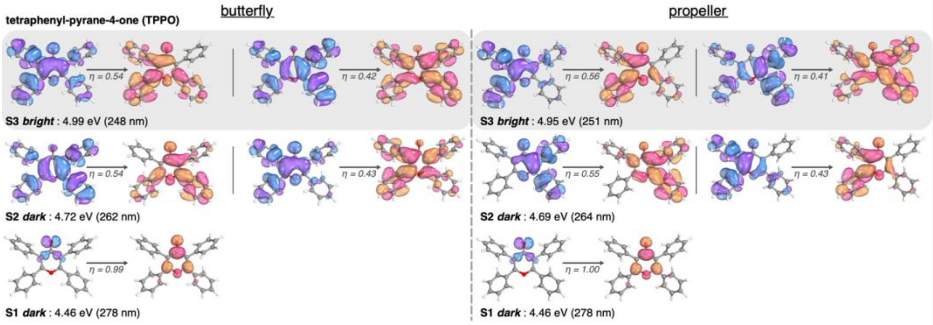

Figure S24. Natural transition orbitals for S1, S2, and S3 states of TPPO in implicit THF at the TD-wB97X-D3/def2-SVP level. The occupation number (η) for the relevant NTO couples is given as well as the energy of the transition.

2.11 REFERENCES

1. Singh, P.; Bhardwaj, A. Mono-, Di-, and Triaryl Substituted Tetrahydropyrans as Cyclooxygenase-2 and Tumor Growth Inhibitors. Synthesis and Biological Evaluation. *J. Med. Chem.* **2010**, *53* (9), 3707–3717.
2. Harding, K. E.; May, L. M.; Dick, K. F. Selective Oxidation of Allylic Alcohols with Chromic Acid. *J. Org. Chem.* **1975**, *40* (11), 1664–1665.
3. Allwohn, J.; Brumm, M.; Frenking, G.; Hornivius, M.; Massa, W.; Steubert, F. W.; Wocadlo, S. Synthese Und Konformationsanalyse von Pyranophanonen Und Pyrylophanium-Verbindungen Mit Intraannularen Substituenten. *J. Für Prakt. ChemieChemiker-Ztg.* **1993**, *335* (6), 503–514.
4. Brouwer, A. M. Standards for Photoluminescence Quantum Yield Measurements in Solution (IUPAC Technical Report). *Pure Appl. Chem.* **2011**, *83* (12), 2213–2228.
5. Neese, F., Wennmohs, F., Becker, U., & Riplinger, C. (2020). The ORCA quantum chemistry program package. *J. Chem. Phys.* **2020**, *152*, 224108-224118.
6. a. Mai, S.; Richter, M.; Heindl, M.; Menger, M. F. S. J.; Atkins, A.; Ruckenbauer, M.; Plasser, F.; Ibele, L.M.; Kropf, S.; Oppel, M.; P. Marquetand, L. G. SHARC2.1: Surface Hopping Including Arbitrary Couplings – Program Package for Non-Adiabatic Dynamics. sharc-md.org (Accessed 2023-01-28): b. Richter, M.; Marquetand, P.; González-Vázquez, J.; Sola, I.; González, L. SHARC: Ab Initio Molecular Dynamics with Surface Hopping in the Adiabatic Representation Including Arbitrary Couplings. *J. Chem. Theory Comput.* **2011**, *7,* 1253– 1258; c. Mai, S.; Marquetand, P.; González, L. Nonadiabatic Dynamics: The SHARC Approach. *Wiley Interdiscip. Rev. Comput. Mol. Sci.* **2018**, *8,* e1370
7. a. Knizia, G. Intrinsic atomic orbitals: An unbiased bridge between quantum theory and chemical concepts. J. Chem. Theory Comput., 2013, 9, 4834-4843. b. Knizia, G., Klein, J. E. Electron flow in reaction mechanisms—revealed from first principles. *Angew. Chem. Int. Ed.*, **2015**, *54*, 5518-5522.
